# Methyl-CpG binding domain 2 (Mbd2) is an epigenetic regulator of autism-risk genes and cognition

**DOI:** 10.1038/s41398-023-02561-9

**Published:** 2023-07-13

**Authors:** Elad Lax, Sonia Do Carmo, Yehoshua Enuka, Daniel M. Sapozhnikov, Lindsay A. Welikovitch, Niaz Mahmood, Shafaat A. Rabbani, Liqing Wang, Jonathan P. Britt, Wayne W. Hancock, Yosef Yarden, Moshe Szyf

**Affiliations:** 1 Department of Molecular Biology, Ariel University, Ariel, Israel; 2 Department of Pharmacology and Therapeutics, McGill University, Montreal, QC Canada; 3 Department of Biological Regulation, Weizmann Institute of Science, Rehovot, 76100 Israel; 4 Department of Neurology and Neurosurgery, McGill University, Montreal, QC Canada; 5 Department of Medicine, McGill University Health Center, Montreal, QC Canada; 6 Department of Biochemistry, McGill University, Montreal, QC Canada; 7 Division of Transplant Immunology, Department of Pathology and Laboratory Medicine, and Biesecker Center for Pediatric Liver Diseases, Children’s Hospital of Philadelphia and Perelman School of Medicine, University of Pennsylvania, Philadelphia, PA USA; 8 Department of Psychology, McGill University, Montreal, QC Canada; 9 Present Address: Department of Neurology, Massachusetts General Hospital, Charlestown, MA 02129 USA; 10 Present Address: Harvard Medical School, Boston, MA 02115 USA

**Keywords:** Epigenetics and behaviour, Molecular neuroscience

## Abstract

The Methyl-CpG-Binding Domain Protein family has been implicated in neurodevelopmental disorders. The Methyl-CpG-binding domain 2 (Mbd2) binds methylated DNA and was shown to play an important role in cancer and immunity. Some evidence linked this protein to neurodevelopment. However, its exact role in neurodevelopment and brain function is mostly unknown. Here we show that *Mbd2*-deficiency in mice (*Mbd2−/−*) results in deficits in cognitive, social and emotional functions. Mbd2 binds regulatory DNA regions of neuronal genes in the hippocampus and loss of *Mbd2* alters the expression of hundreds of genes with a robust down-regulation of neuronal gene pathways. Further, a genome-wide DNA methylation analysis found an altered DNA methylation pattern in regulatory DNA regions of neuronal genes in *Mbd2−/−* mice. Differentially expressed genes significantly overlap with gene-expression changes observed in brains of Autism Spectrum Disorder (ASD) individuals. Notably, downregulated genes are significantly enriched for human ortholog ASD risk genes. Observed hippocampal morphological abnormalities were similar to those found in individuals with ASD and ASD rodent models. Hippocampal *Mbd2* knockdown partially recapitulates the behavioral phenotypes observed in *Mbd2−/−* mice. These findings suggest that Mbd2 is a novel epigenetic regulator of genes that are associated with ASD in humans. Mbd2 loss causes behavioral alterations that resemble those found in ASD individuals.

## Introduction

Epigenetic modifications of the genome are long known to play a crucial role in normal brain function including a wide range of neuropsychological processes as well as in neuropsychological disorders [1]. The most studied epigenetic modification is DNA methylation, the addition of a methyl group to the DNA on a cytosine predominantly in a CpG dinucleotide context. Nevertheless, non-CpG methylation does occur especially in non-dividing cells like neurons, is increased during postnatal brain development and might play an important role in regulating brain function^2^. DNA methylation in promoters and other regulatory regions suppresses gene expression by interfering with transcription factors and transcription machinery binding [2]. An additional mechanism involves recruitment of members of a methylated-DNA binding proteins family (MECP2 and MBD1-6) which share a Methyl-CpG Binding Domain (MBD) [3, 4]. MECP2 and MBD2 were shown to recruit chromatin repressive complexes to genes and thus cause alterations in histone modification and silencing of gene expression [5–7]. These proteins are highly expressed in brain tissues [8, 9].

The most extensively studied MBD protein is MeCP2 since mutations and duplications of this gene cause Rett syndrome and MECP2 duplication syndrome respectively [10, 11]. Some studies on the role of MBD1 suggest it has a role in neurodevelopment and neurodevelopmental disorders like autism [12]. In contrast, little is known about the roles of other MBD proteins in the brain.

Several studies allude to possible involvement of MBD2 in neuropsychiatric disorders, however none of these studies support yet genetic association or linkage of MBD2 to neuropsychiatric disorders. An increased MBD2 DNA binding on the promoters of *BDNF*, *GAD1* and *RELN* genes was observed in post-mortem brains of schizophrenia and bipolar disorder patients [13]. Rare nonsynonymous de-novo mutations in *MBD2* were identified in ASD [14–16]. Several studies found copy number variants (CNV) at and around the genomic position of the *MBD2* gene (18q21.2) in ASD individuals (both deletions and duplications; see SFARI-CNV database: https://gene.sfari.org/database/cnv/). However, in these cases the CNVs span also other genes associated with neurodevelopmental disorders, including *TCF4* (Pitt-Hopkins syndrome), *ASXL3* (Bainbridge-Ropers syndrome) and *SETBP1* (ID syndrome MIM #616078 and Schinzel-Giedion syndrome); thus confounding the role of Mbd2 deletions in the etiology of ASD.

In rat pups of low maternal-care mothers there is reduced hippocampal Mbd2 expression compared to offspring of high maternal-care mothers which corelates with reduced glucocorticoid-receptor expression and elevated stress [17]. However, to date the mechanisms by which Mbd2 affect gene-expression and ultimately brain function and behavior are unclear.

Here, we directly assessed the role of *Mbd2* in behavior using a knockout mouse model (*Mbd2−/−*). A comprehensive behavioral battery found cognitive, social and emotional deficits in Mbd2−/− mice. Several lines of evidence link ASD-associated MBD proteins to hippocampus development and function, including MeCP2 [18–20] and Mbd1 [12, 21]. Since the behavioral abnormalities pointed to hippocampal functions, we examined the molecular footprints of *Mbd2* in the hippocampus. We applied unbiased genome-wide approaches. Using ChIP-seq we found that Mbd2 binds methylated and unmethylated CpGs on and around many neuronal genes in the hippocampus as well as other genomic loci. Many of these binding peaks were in sites of bivalent histone marks, a regulatory mechanism which is thought to represent a reversibly repressed state allowing for epigenetic plasticity [22]. Loss of Mbd2 binding in *Mbd2−/−* mice led to down-regulation of neuronal-gene expression. In contrast, upregulated genes in *Mbd2−/−* mice were associated mostly to homeostasis and cell maintenance gene pathways. *Mbd2*-deficiency led to increased methylation on promoters and enhancers of neuronal genes, suggesting that Mbd2 affects DNA methylation status at neuronal genes and hence hippocampal genome functions. Furthermore, we found that differentially expressed genes were highly overlapped and correlated to differentially expressed genes found in ASD individuals’ brain. Downregulated genes were highly enriched for ortholog ASD risk-genes implying an important role for Mbd2 in neurodevelopment and neuropsychiatric disorders.

## Materials and methods

For additional details on methods, including behavioral procedures, lentivirus infusion, bioinformatic analyses, molecular and histological procedures and statistics please see the supplemental online materials.

### Animals

The *Mbd2−/−* line was created in the lab of Dr. Adrian Bird [23]. The embryos of heterozygous mice were kindly gifted by Dr. Brian Hendrich from the University of Cambridge which were bred at McGill University animal facility. Mice were housed in groups (3–5/cage) with 12-h light/dark cycles under conditions of constant temperature (23 °C) and humidity (50%), and free access to food and water. All procedures were carried out in accordance with the guidelines set down by the Canadian Council of Animal Care and were approved by the Animal Care Committee of McGill University.

Previous studies [23] found that *Mbd2−/−* dams show impaired maternal care and nurturing behavior, which in turn results in slower weight gain of the pups. These phenomena were not observed in heterozygote mice [23].

Therefore, all littermates were bred from heterozygote parents to avoid confounding effect of maternal behavior. All ex-vivo experiments were conducted on 10–12 weeks old mice.

### Chromatin-immunoprecipitation followed by sequencing (ChIP-Seq)

Mice were sacrificed and bi-lateral hippocampi were rapidly isolated, flash frozen and stored at−80 °C for later analysis. For Chromatin-Immunoprecipitation, hippocampi from two independent groups of wild-type littermates and two independent groups of Mbd2*−/−* littermates (*n* = 6–8) were pooled, homogenized in 1 X PBS including 1% formaldehyde, and the homogenates were kept for 10 min at 25 °C. Cross-linking reactions were stopped by the addition of glycine (125 mM) for 10 min at 25 °C. Fixed chromatin samples were then homogenized in cell lysis solution (PIPES 5 mM (pH 8), KCl 85 mM, NP40 0.5%) and centrifuged for 5 min at 3000 rpm, 4 °C. Pellets were resuspended in RIPA-light solution (NaCl 150 mM, SDS 0.3%, Tris-HCl 50 mM (pH 8)) and sonicated using a Covaris E220. Sonicated chromatin samples were then centrifuged for 15 min at 14000 rpm, 4 °C. Pellets were resuspended in 1 ml of RIPA-light solution. Chromatin samples were pre-cleared with 50 µl of Dynabeads protein-G (Life Technologies, Ottawa, ON) pre-blocked with BSA and incubated overnight at 4 °C with an anti-Mbd2 (IP-grade, Epigentek-A1007, Burlington, ON) antibody. Input control was treated the same way except for not adding an anti-Mbd2 antibody to the solution. Antibodies and chromatin were then mixed with 100 µl of Dynabeads protein-G for 3 hours at 4 °C. The beads were then washed with RIPA-light solution, then with wash-solution (Tris-HCl 100 mM (pH 8), LiCl 500 mM). Protein–DNA complexes were eluted from the beads, de-cross-linked, treated with proteinase K and purified. The DNA concentration was determined by fluorometry on the Qubit system (Invitrogen, Ottawa, ON). A total of 10–12 ng DNA were used for library preparation. The immunoprecipitated- and input-DNA were sheared a second time with the Covaris E220 instrument in 53 µl reaction volume (duty factor 10%, Pic Incident Power 175, Cycles per burst 200, time 360 sec) to obtain fragments in the size range of 150 bp followed by purification with AMPure XP beads (×1.8 v/v) (Beckman Coulter A63881, Indianapolis, IN). Purified DNA was resuspended in 45 µl elution buffer. Libraries of the chromatin immunoprecipitated and input DNA fragments were prepared using the Tru Seq DNA Low Throughput Protocol (Illumina). PCR enrichment of ligation products was performed using the Illumina Primer Cocktail; 15 PCR cycles were performed for ChIP libraries and 10 cycles for the input. The libraries were purified using AMPure XP beads ×1.0 v/v. Quality of libraries was validated by 260 nm absorbance measurement, quality control on HSdna chip (Agilent Bioanalyzer: size of libraries around 275 bp) and quantification by Q-PCR with Kappa Library Quantification kit for Illumina Sequencing Platforms (KAPPA Biosystems). The DNA concentration of the different sequencing libraries was from 40 to 500 nM. Clusters (13.5 pM) were generated using TruSeq PE Cluster Kit v3, for cBot protocol, followed by 50 bp either single or pair--end sequencing, on an Illumina HiSeq2000, per the manufacturer recommendations. Bioinformatic Analysis of the ChIP-Seq Data is detailed in the supplemental materials.

### RNA-sequencing and data analysis

DNA and RNA from 8 to 10 mice were isolated and purified with AllPrep-DNA/RNA/miRNA-universal kit (Qiagen, Montreal, QC) and concentrations were determined by fluorometry on the Qubit system (Invitrogen, Ottawa, ON). Ribosomal RNA was removed using the Ribominus kit (Invitrogen). cDNA libraries (4 samples/group, hippocampi from 2 mice were pooled per sample) were generated and sequenced using an Illumina Hiseq2500 (100 bp pair-end), as instructed by Illumina’s RNA-seq protocols. Reads were deduplicated and aligned to the mouse reference transcriptome (mm9) with STAR aligner [24]. Differential expression was analyzed using DeSeq2 (FDR < 0.05) [25].

### Human brain transcriptomic data analysis

The PsychENCODE Consortium [26] published RNA sequencing data from brain samples of 1695 individuals with ASD, schizophrenia, bipolar disorder and controls. We used this database to create gene lists of known human genes, which were differentially expressed in human brains (FDR < 0.05). Overlaps between these human genes and differentially-expressed genes in *Mbd2*−/− mice hippocampi were analyzed. Next, we analyzed the correlations between the magnitude of changes (expressed as fold change) in gene expression between species for each disorder.

### Human genetics data analysis

ASD-risk gene list was extracted from Sanders et al. [27] (65 genes; *n* = 10,220; FDR < 0.1). Additionally, we extracted data from the SFARI-GENE project (gene.sfari.org) that contains of ASD-associated gene list based on human and animal model studies. Genes are grouped according to criteria for the strength of evidence for each gene (a total of 970 genes as of 23 November 2017). We excluded 19 genes categorized in category 6- for genes studied in human cohorts and findings suggest against a role in autism, having a total of 951 ASD-associated genes from this database. We assessed the overlap between ASD-risk genes from each of the databases to the ortholog mouse genes which were differentially expressed in *Mbd2−/−* mice. Significance was determined by a hypergeometric test.

### Capture bisulfite sequencing and DNA methylation mapping

DNA was extracted from two groups of littermate mice (8–10 mice/group per genotype). Each group was pooled and sequenced using the SeqCap Epi Enrichment System (Roche NimbleGen, Laval, QC) for targeted-bisulfite sequencing of promoters and enhancers as we described before [28, 29]. Mouse target probes (mm9) were custom-designed based on H3K4me1 and H3K4me3 signals from mouse public ChIP-seq data. Biotinylated target probes were designed for both strands of bisulfite-converted genomic DNA. Bisulfite-treated genomic DNA was ligated to methylated adapters, hybridized to the biotinylated oligonucleotide probes followed by a series of washes of off-target DNA sequences and unbound DNA. Isolated DNA then underwent PCR amplification and sequenced on Illumina Hiseq2000 with pair-end 50 bp reads and a technical repeat with 125 bp pair-end reads was performed as well.

## Results

### Mbd2 is required for cognitive, social, and emotional behaviors

**Fig. 1:**
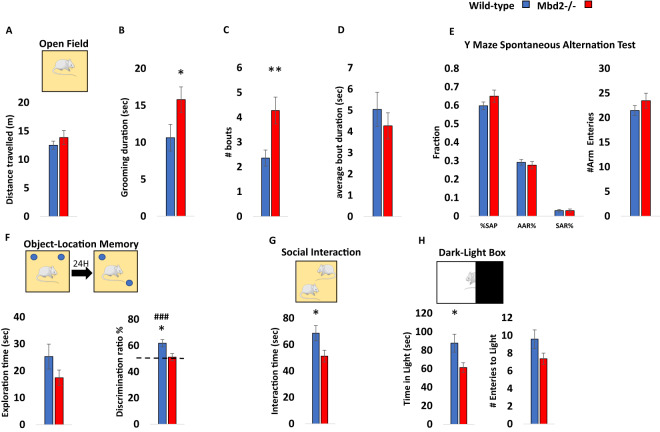
Behavioral effects of *Mbd2* deficiency. **A** Locomotion in the Open-field box was assessed for 5 min. *n* = 14 wild-type, *n* = 15 mMbd2*−/−*. **B** Self-grooming during open field. (Two-way ANOVA, main effect of genotype F(1,28) = 5.2, *p* = 0.0304). **C** Number of self-grooming bouts. (Two-way ANOVA, main effect of genotype F(1,28) = 9.62, *p* = 0.0044). **D** Average self-grooming bout duration. **E** Y-maze spontaneous alteration was not affected in *Mbd2−/−* mice (right). SAP-Spontaneous Alteration Performance, AAR-Alternate Arm Return, SAR-Same Arm Return. Exploration is expressed as number of arm entries (right). *n* = 17 wild-type, *n* = 17 *Mbd2−/−*. **F** Exploration time during object-location memory training (left) and discrimination ratio in object-location memory test (right). Discrimination ratio was significantly lower in *Mbd2−/−* mice (Two-way ANOVA, main effect of genotype F(1,24) = 5.05, *p* = 0.0341; *n* = 13 wild-type, *n* = 15 *Mbd2−/−*). **G** Social interaction. Mice were introduced to a novel mouse for 5 min and interaction time was recorded. (Two-way ANOVA, main effect of genotype F(1,29) = 5.02, *p* = 0.0329; *n* = 17 wild-type, *n* = 16 *Mbd2−/−*). **H** Time spent in the light compartment of the Dark-Light Box (left) (Two-way ANOVA, main effect of genotype F(1,29) = 5.3, *p* = 0.0287; *n* = 17 wild-type, *n* = 16 Mbd2*−/−*) and number of entries to the light side (right). Data are presented as mean ±SEM *p < 0.05, **p < 0.01 for wild-type vs *Mbd2−/−*. ### *p* < 0.005 for wild-type over chance (50%) exploration (one-sample t-test). Since no main effect for sex was observed in any of the tests (for full data see Figs. S1–S3) behavioral data from males and females were collapsed within genotype for clarity.

Previous studies on *Mbd2−/−* mice behavior found impaired maternal behaviors such as suboptimal feeding and delayed pup retrieval [23]. Hypoactivity, impaired nest-building behavior and mild spatial learning and memory impairments were found in male Mbd2*−/−* mice [30]. We further tested whether *Mbd2* is involved in behavior using additional behavioral tests. We tested both male and female mice and found no significant effect of sex for any of the behaviors measured (two-way ANOVAs, *p* > 0.05 for main effect of sex and for interactions in all cases, Figs. S1–S3, with the exception of a significant main effect for sex on grooming bout length (Fig. S2C)). We therefore, grouped both males and females for subsequent experiments. There was no change in general locomotion and exploration in an open-field box (Fig. 1A, Fig. S4) However, we observed a significantly increased self-grooming time and number of grooming bouts in Mbd2*−/−* mice (Fig. 1B–D). Several forms of memory were tested as well. Short-term working memory was assessed by spontaneous alternations in Y-Maze and was found to be intact in *Mbd2−/−* mice. Also, the number of arm entries did not differ between groups suggesting normal exploration behavior in *Mbd2−/−* mice (Fig. 1E). In contrast, *Mbd2−/−* mice showed impaired memory-retention in the long-term object-location memory test and failed to explore the object in the novel location beyond chance levels (Fig. 1F). A previous study [30] did not find significant deficits in social behavior in *Mbd2−/−* mice using a three-chamber test which is a common test for sociability in mice. However, some mouse models for neurodevelopmental disorders show normal or near-normal behavior in the three-chamber test while showing reduced sociability in the social-interaction test [31–33]. These data suggest that the social-interaction test is either more sensitive and/or measures different aspects of sociability than the three-chamber test as it allows direct physical interaction and exploration between the mice in the arena. We therefore used the social-interaction test and found that *Mbd2−/−* mice exhibited reduced social-interaction time (Fig. 1G). We also measured anxiety-like behavior in the dark-light box and found that *Mbd2−/−* mice spent less time in the light compartment which indicates higher anxiety levels (Fig. 1H). Our findings support the hypothesis that Mbd2 is involved in regulating cognitive, social and emotional behaviors.

### Landscape of Mbd2 binding in the hippocampus

**Fig. 2:**
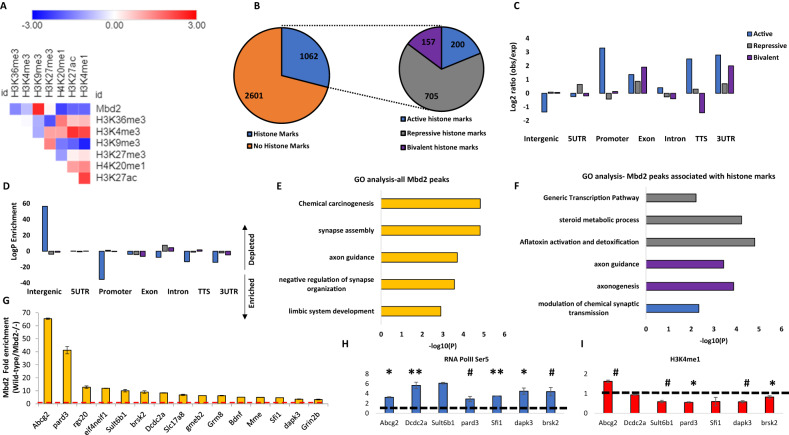
Landscape of Mbd2 binding delineated by ChIP-seq. **A** Heatmap of the overlap between Mbd2 peaks and the following histone-marks in the hippocampus: H3K4me1, H3K4me3, H3k2me3, H3K27ac, H39Kme3, H3K36me3 and H4K20me1. **B** Analysis of the number of Mbd2 peaks located on histone-marks. **C** Co-occupancy enrichment analysis and **D** significance level of Mbd2 and histone-marks. **E** Pathway analysis enrichment of Mbd2 binding peaks (Top 5 pathways are presented). **F** Pathway analysis enrichment of Mbd2 binding peaks co-occupied with histone marks. **G** Q-ChIP validation of Mbd2 peaks. **H** Q-ChIP for RNApolII (SP5) and **I** H3K4me1. For Q-ChIP of Mbd2, RNApolII(SP5) and H3K4me1 pool of 5–6 mice analyzed by triplicate/group). Data are presented as mean ±SEM # *p* < 0.1, *p < 0.05, **p < 0.01 (T-test). Dashed lines represent Mbd2*−/−* binding levels. TTS Transcription Termination Site. UTR untranslated region.

We focused our analysis on the hippocampus since the behaviors affected in our study are known to involve hippocampal functions [34–38]. Additionally, other members of the MBD protein family were shown to have an important role in hippocampal function [12, 18, 39, 40]. We performed Mbd2 ChIP-sequencing on wild-type and Mbd2*−/−* animals to identify Mbd2 binding sites. We identified 2782 Mbd2 peaks annotated to 461 genes (FDR < 0.05, Fig. S5 for examples of MBD2 occupancy across relevant genomic regions). As expected, Mbd2 binds mostly to CpG-containing and GC-rich DNA regions (Χ^2^ = 123.49; df=1, *p* = 2.2E-16 and Χ^2^ = − 7168.2; df=1, *p* = 2.2E-16, respectively). De-novo motif discovery found the transcription-factors E2F8 and NFAT5 to be highly enriched in Mbd2 binding-peaks (Fig. S6A). NFAT family members were also enriched in known motif-enrichment analysis (Fig. S6B). The E2F and NFAT families have been reported to have a role in neurogenesis, and brain development [41–44]. We also compared our data to publicly available ChIP-Seq data of the mouse hippocampus histone-marks [45]. As expected, Mbd2 bound overall to regions that bind more repressive histone-marks (mostly H3K9me3) than to regions that bind active histone-marks (Fig. 2A, B). However, a subset of the peaks was bound to regions that had both active and repressive histone-marks. This might imply that either Mbd2 binds sites that are bivalently marked in the same cell or that Mbd2 binds DNA regions that have different chromatin states in different hippocampal cell-populations (Fig. 2C, D). Mbd2 co-occupancy with histone-marks on promoters, 3’-UTRs, TTS and exons was enriched for active histone-marks and depleted from repressive histone-marks consistent with an activating role in these genomic features (Fig. 2D). Exons and intergenic regions showed mildly enriched Mbd2 co-occupancy with repressive histone-marks (Fig. 2D). Pathway-analysis by GO-enrichment revealed enrichment of Mbd2 binding at several neuronal- and brain-related pathways including synapse assembly and axon-guidance (Figs. 2E and S7). GO-analysis of Mbd2 peaks associated with histone-marks found that Mbd2 peaks associated with active and bivalent histone marks are enriched for brain-related pathways including: axon guidance, axonogenesis and modulation of chemical synaptic transmission, thus further supporting a role for Mbd2 in neuronal development and function (Fig. 2F).

We selected several peaks of Mbd2 that were associated with promoters or enhancers in neuronal-related genes for further validation and for determining the impact of Mbd2 binding loss on transcription initiation (Table S1). Quantitative-ChIP-PCR confirmed Mbd2-binding to these regions and loss of binding in *Mbd2−/−* mice (Fig. 2G). We then determined whether loss of Mbd2 binding in promoters or enhancers in *Mbd2−/−* mice affects transcription onset. *Mbd2*-deficiency resulted in reduced transcription onset since occupancy of RNA-polymerase phosphorylated on Serine5; (RNApolII(SP5)) the form found on promoters upon transcription-initiation [46, 47] was reduced in *Mbd2−/−* mice. This was associated with increased abundance of histone-mark (H3K4me1) marking enhancers in *Mbd2−/−* hippocampi (Fig. 2H, I). H3K4me1 modification marks active as well as inactive enhancers [48, 49]. H3K4me1 peaks that are flanking the TSS were shown to exhibit a peak-valley-peak pattern. The valley usually overlaps with transcription-initiation and RNApolII(SP5) peaks [50] reflecting a nucleosome-free zone and thus reduced histone presence and reduced signal for the histone-marks, which is prerequisite for transcription-initiation [51]. In transcriptionally inactive genes H3K4me1 peaks cover the entire TSS forming one continuous TSS-centered peaks that overlap with the RNApolII(SP5) peaks. The increase of H3K4me1 concurrently with reduction of RNApolII(PS5) binding at the same position in *Mbd2−/−* mice is consistent with inhibition of transcription-onset in response to *Mbd2-*deficiency. These data suggest that Mbd2 is involved in activation of transcription turn-on in these neuronal-specific promoters.

### Mbd2 is required for expression of neuronal-genes

**Fig. 3:**
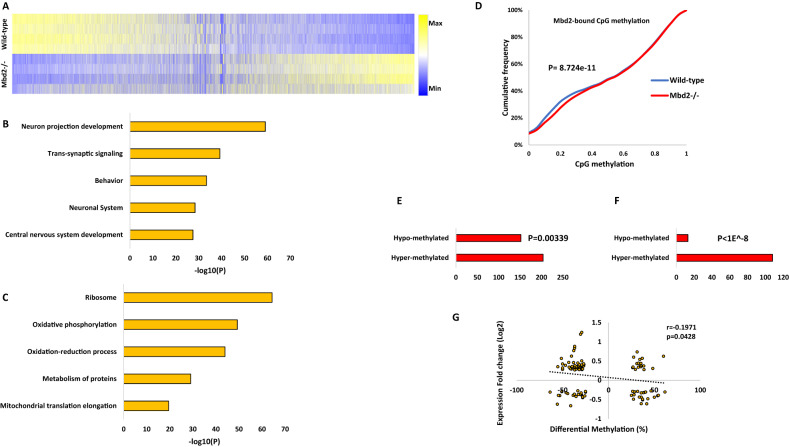
Effect of Mbd2 deficiency on gene expression and DNA methylation. **A** Heatmap of all transcribed genes in *Mbd2−/−* and wild-type mice sorted by log2 fold-change. **B** Pathway analysis enrichment of downregulated genes (Top 5 pathways are presented). **C** Pathway analysis enrichment of upregulated genes (Top 5 pathways are presented). **D** A cumulative distribution of methylation levels of regulatory DNA regions bound by Mbd2 demonstrating hyper-methylation in *Mbd2−/−* hippocampus (K-S test). **E** Number of differentially methylated CpGs (with at least 10% change in methylation and *p* < 0.001) in Mbd2-bound annotated genes showing that more CpGs became hyper-methylated then hypo-methylated (*p* = 0.00339, binomial test). **F** Number of differentially methylated CpGs (with at least 10% change in methylation and *p* < 0.001) located within Mbd2-peaks (*p* < 1E-8, binomial test). **G** Pearson correlation between differential promoter DNA-methylation and gene-expression in *Mbd2−/−* hippocampi.

To further elucidate the role of Mbd2 in hippocampal gene-expression we delineated the transcriptomes of wild-type and *mbd2−/−* mice. We found 2907 differentially-expressed genes (FDR < 0.05), of which 1590 genes were upregulated, and 1317 genes were downregulated in *Mbd2−/−* mice (Figs. 3A and S8). GO-enrichment analysis found a robust down-regulation of neuronal-related pathways such as neuron-projection development, trans-synaptic signaling and behavior (Fig. 3B) which were highly organized in clusters based on clustering analysis of the GO-networks (Fig. S9A, B). In contrast, upregulated genes were enriched for homeostasis and metabolism-related pathways (Fig. 3C). GO-analysis revealed poor clustering of the upregulated genes in these pathways (Fig. S10A, B). Next, we analyzed the subsets of genes that were annotated to Mbd2 binding peaks and also showed a significant change in their transcription levels. We did a pathway enrichment analysis for genes that were both annotated to Mbd2 ChIP peaks and were significantly up- or down-regulated at their transcription levels. In line with our other findings, we found 16 genes with Mbd2 peaks and downregulated mRNA levels were enriched for the GO-terms: “Cognition” *p* = 7.94E-05 and “Regulation of growth” *p* = 0.012. We also found 20 genes with Mbd2 peaks and upregulated mRNA levels that were enriched for the GO-term: “Membrane trafficking” *p* = 0.01.

### The impact of *Mbd2* depletion on the DNA methylation landscape

MBD proteins are “readers” of DNA methylation and bind specifically to methylated DNA [3, 52]. However, it is possible that they also play a role in maintaining DNA methylation/demethylation landscapes as has been previously proposed. Previous studies in T-regulatory cells using *Mbd2−/−* mice have suggested that *Mbd2* depletion causes hypermethylation of regulatory regions which in turn results in downregulation of gene expression [53]. Therefore, we tested whether M*bd2*-deficiency would alter the hippocampal DNA methylation landscape. We mapped with targeted-bisulfite-sequencing the methylation state of regulatory DNA regions (promoters and enhancers, see methods) in the hippocampi of M*bd2−/−* and wild-type mice at a single-base resolution. An analysis of methylation level distribution across all sequenced CpGs revealed that Mbd2 binding regions defined in wild-type mice by ChIP-seq were hypermethylated in *Mbd2−/−* mice (Fig. 3D). Hypermethylation in response to *Mbd2* depletion was observed also in a genome-wide scale analysis which examined DNA outside Mbd2-bound regions (Fig. S12C). The fact that changes in DNA methylation occur in regions outside of Mbd2 peaks suggests that *Mbd2*-deficiency could affect DNA methylation indirectly. We, therefore, examined our RNA-seq data for expression levels of methylated-CpG readers and modifiers. We found a significantly reduced expression of *Tet2*, *Dnmt3a*, *Mbd1* and *Mecp2* in *Mbd2−/−* mice (Fig. S11). The reduced expression levels of enzymes that promote de-novo DNA methylation (*Dnmt3a*) and de-methylation (*Tet2*), together with the reduced levels of methyl-CpG binding proteins (*Mbd1* and *Mecp2*) possibly contributed to changes observed in the landscape of DNA methylation in *Mbd2−/−* mice. Taken together these data suggest that loss of *Mbd2* affects DNA methylation in both directions. An analysis of the sequence properties of Mbd2-dependent DNA methylation suggests that *Mbd2*-deficiency results in increased methylation of low to intermediate methylated CpGs (10–40% methylation, Fig. 3D) but highly methylated CpGs ( > 40% methylation, Fig. 3D) are unaffected as expected if Mbd2 prevents hypermethylation. Partially methylated genes represent a group of genes that are heterogeneously methylated and possibly heterogeneously expressed across hippocampal nuclei. Mbd2 might be regulating the state of methylation of these genes.

We also analyzed differential-methylation at single CpG resolution. At this resolution differential-methylation analysis (at least 10% difference, FDR < 0.05; Fig. S12A) revealed 323 differentially-methylated CpGs, with 133 hypo-methylated CpGs (annotated to 103 genes) and 190 hyper-methylated CpGs (annotated to 119 genes) (differential-methylation data (*p* < 0.001) is detailed at Table S2). This finding also supports a significant overall hypermethylation in *Mbd2−/−* mice hippocampus (*p* = 0.0018, binomial-test). For pathway-analysis, to obtain a larger number of terms for the pathway analysis, we applied a more lenient significance cut-off for our data (*p* < 0.001) resulting in 3005 differentially methylated CpGs (1519 hypo-methylated and 1486 hyper-methylated). Next, we focused the analysis on CpGs located on promoters ( ± 1000 bp from TSS). We found 494 hypo-methylated CpGs located in 460 gene promoters and 476 hyper-methylated CpGs located in 434 gene promoters (Fig. S12A). Pathway analysis for differentially methylated gene-promoters revealed adrenergic receptor signaling and sodium- and metal- ion transports as the top three hyper-methylated pathways (-log10*p*-values: 4.86, 4.16 and 3.42, respectively). In contrast, the top hypo-methylated pathways were not specifically related to neurons or brain functions, although neuronal ion-channel clustering and positive regulation of sodium ion-transport pathways were enriched (-log10p-values: 3.289 and 3.280, respectively) (Fig. S12E, F). Overall, this analysis suggests an enrichment of hyper-methylated genes related to neuronal gene pathways in *Mbd2−/−* mice hippocampi.

Next, we assessed how binding of Mbd2 affects DNA methylation at single CpG resolution in Mbd2-bound genes. We analyzed the changes in DNA methylation levels in knockdown animals for CpGs (with at least 10% change and *p* < 0.001) annotated to Mbd2-bound genes and found significantly more genes had hyper-methylated CpGs (Fig. 3E). When analyzing only the subset of these CpGs which were located directly at Mbd2 binding peaks we found in almost all cases ( ~ 90%) hyper-methylation of these CpGs (Fig. 3F). This is consistent with a role for Mbd2 binding in maintaining a hypomethylated state.

To assess the relation between promoter DNA methylation and gene expression, we determined the correlation between methylation and expression. We found a significant linear inverse correlation between promoter DNA methylation and gene expression (Fig. 3G), supporting a role for DNA methylation in gene repression in the hippocampus. Unexpectedly, a subset of differentially methylated CpGs in promoters showed positive correlation between expression and methylation. We explored this finding by analyzing DNA motifs around these CpGs. While CpGs with inverse correlation (*n* = 66) had no significantly enriched motifs; CpGs with positive correlation (*n* = 40) had 3 enriched motifs (*p* values = 0.015–0.025). These enriched motifs were highly similar to known motifs of the KLF and SP transcription-factor families with top similarities to: KLF12, KLF14, SP3, and SP8 (Fig. S12D). The KLF and SP transcription factors are known to prefer binding to methylated-DNA (KLF) or to be unaffected by DNA methylation (SP) [54]. This finding suggests that genes whose methylation status does not correlate inversely with their expression, are regulated by transcription-factors that are not inhibited by DNA methylation.

We also validated our DNA methylation analysis results by targeted sequencing of bisulfite-converted PCR amplicons of nine Mbd2-binding regions each of which annotated to a different gene (Table S5)). Methylation levels in these Mbd2-bound regions strongly correlated with the levels obtained in the genome-wide capture sequencing analysis (Fig. S13A, B). Loss of *Mbd2* promoted bi-directional alterations in DNA methylation status with enhanced hyper-methylation in Mbd2 binding regions, as observed earlier for the genome-wide analysis (Fig. S13C, D). Overall, there was a significant hyper-methylation in *Mbd2−/−* samples (Fig. S13E) as was observed in the genome-wide capture-sequencing.

### Differentially expressed genes in *Mbd2−/−* mice are homologs of differentially expressed genes in ASD individuals’ brains and ASD risk genes

MBD proteins other than Mbd2 including MeCP2, Mbd1, Mbd5 and Mbd6 were previously proposed to be involved in ASD [11, 12, 39, 40, 55] and the presentation of behavioral abnormalities in *Mbd2−/−* mice described above resembles some of the observed symptoms of ASD. Therefore, we sought to explore the potential relation between our gene-expression findings and those found in post-mortem brains from neurodevelopmental disorders patients. We compared our data to the recently released cortex transcriptomic data from the psychENCODE consortium [26] for ASD, schizophrenia and bipolar disorder.

**Fig. 4:**
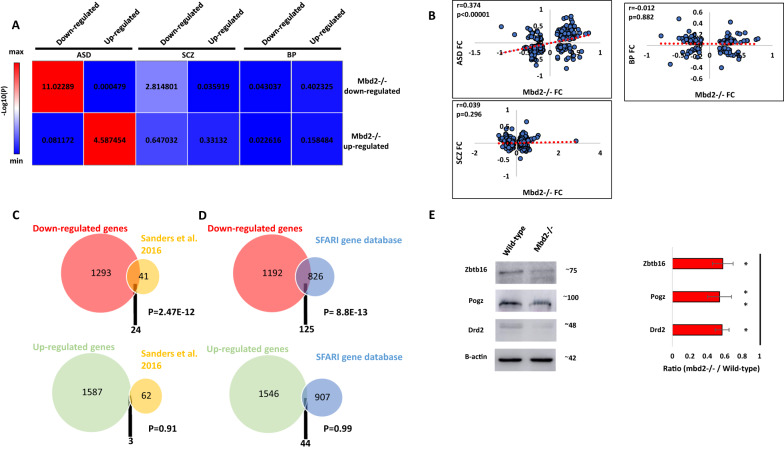
Overlap between *Mbd2−/−* mice hippocampal transcriptome, human brain transcriptomes in psychiatric disorders and ASD-risk genes. **A** Heatmap of log10(p-values) of differentially expressed gene overlap between *Mbd2−/−* mice and ASD, schizophrenia and bipolar disorder human brain. Numbers within the heatmap indicate -log10(p-values). **B** Pearson’s correlations of fold-changes in gene-expression levels between *Mbd2−/−* mice and ASD (*n* = 286 genes), schizophrenia (*n* = 686 genes) and bipolar disorder human brain (*n* = 137 genes). **C** An overlap analysis revealed a significant intersection between human ASD high risk genes and downregulated (Top) but not upregulated (Bottom) genes in *Mbd2−/−* hippocampi. **D** An overlap analysis revealed a significant intersection between human ASD risk genes based on the SFARI Gene Project and downregulated (Top) but not upregulated (Bottom) genes in *Mbd2−/−*. (Hypergeometric tests). **E** Western blot analysis showing reduced protein levels of Drd2, Pogz and Zbtb16 in *Mbd2−/−* mice hippocampi. Proteins molecular weights as determined using molecular weight markers are indicated. Note: circle sizes are not to scale.

We found a significant overlap between differentially regulated genes in Mbd2*−/−* mice and individuals with ASD (Fig. 4A). We also found a weak, though significant, overlap between downregulated (but not upregulated) genes in Mbd2*−/−* mice and downregulated gene in schizophrenia patients (Fig. 4A). We did not find any significant overlap with bipolar disorder differentially expressed genes (Fig. 4A). We also found a significant positive correlation of gene-expression fold-change levels between Mbd2*−/−* mice and ASD individuals but not between Mbd2*−/−* mice and schizophrenia or bipolar-disorder patients (Fig. 4B).

Next, we compared our gene-expression data with that of two human ASD risk-genes databases: a study by Sanders et al. [27]and the SFARI-gene database. We found a robust overlap between ASD-risk genes and downregulated genes in *Mbd2−/−* mice. Twenty-four ( ~ 37%) out of 65 ASD-risk genes found by Sanders et al. [27] were downregulated in *Mbd2−/−* mice (Fig. 4C and Table S3). In contrast, only 3 genes from the 65 gene list were upregulated in *Mbd2−/−* mice. The 24 commonly downregulated genes in *Mbd2−/−* mice and humans also formed a significant protein-protein interaction network (Fig. S14A) with some of the top GO pathway annotations related to “regulation of biological and cellular-processes” and “nervous-system development” (Table S4).

Next, we compared our RNA-seq results to the SFARI-gene list of ASD-associated genes which contains all known human genes associated with ASD. Here again, out of 951 human genes associated with ASD, 125 were downregulated (Fig. 4D and Table S3) while only 44 were upregulated in *Mbd2−/−* mice (Fig. 4D and Table S3). Analysis of Mbd2-regulated ASD-associated gene lists revealed significant protein–protein interactions enrichments. The 125 downregulated genes which appear also in the SFARI ASD-associated gene-database projected into a significantly enriched protein-protein network (Fig. S15A) with “nervous-system development” and ”behavior” as the most enriched GO-pathways within the network (Table S4). The 44 upregulated genes that appear also in the SFARI ASD-associated gene-database projected into a significantly enriched protein-protein network (Fig. S15B) with “dendritic spine morphogenesis” and “modulation of excitatory postsynaptic potential” as the most enriched GO-pathways within the network (Table S4).Taken together, these transcriptomic and genomic cross-species comparative analyses suggest that Mbd2 might serve as an upstream regulator to many ASD-associated genes. This is in line with previous observations that showed regulatory roles for Mbd2 in liver, breast and prostate cancer genes in cancer cell lines [56–58], NGFIA in hippocampal cells [17] and *Foxp3* gene [53] in regulatory T-cells. We further analyzed the protein levels of few members of the protein-protein network of ASD-risk genes and found reduced protein levels of these genes in *Mbd2−/−* mice (Fig. 4E) which agrees with the mRNA reduction we observed before.

Morphological changes in the hippocampus were observed. We found increased neuron count in the CA2 region of the hippocampus, increased neuronal soma size in CA3 and reduced CA1 thickness (Fig. S16). We also found a negative correlation between neuron soma size and neuron count (for CA1 and when all regions’ data was collapsed, Fig. S17) and between neuron soma size and thickness (for CA2, Fig. S17). These changes indicate an increased neuronal density and reduced thickness in the hippocampus of *Mbd2−/−* mice, phenomena which were previously reported in ASD rodent models and human subjects [59–61]. Increased neuronal density was also observed in the cortex of children with autism [62].

### Hippocampal Mbd2 down-regulation impaired cognitive and emotional behaviors

**Fig. 5:**
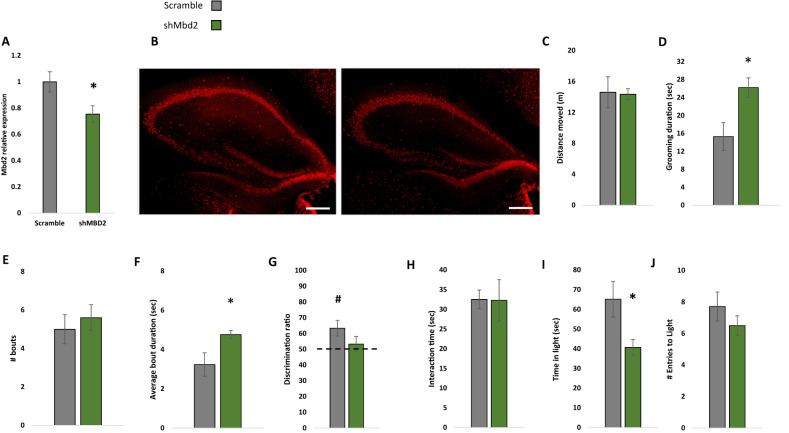
Virus-mediated Mbd2 down-regulation. **A** shMbd2 viral construct reduces *Mbd2* mRNA. Mbd2 mRNA levels were reduced in the hippocampus from shMbd2-infused mice compared with scramble control-infused mice. Scramble *n* = 6, sh*Mbd2* *n* = 4 (T test). **B** Representative micrograph of Mbd2 immunolabeling in hippocampal slices from Scramble- and sh*Mbd2*- infused mice. Scale bar: 200 μm. **C** Locomotion in Open-field box. **D** Self-grooming during open field. **E** Number of self-grooming bouts. **F** Average self-grooming bout duration. **G** Discrimination ratio in object-location memory test. **H** Social interaction test. **I** Time in the light compartment of the Dark-Light Box and **J** number of entries to the light side. Data are presented as mean ±SEM *p < 0.05, for wild-type vs *Mbd2−/−* (t-test). # *p* < 0.05 for wild-type over chance (50%) exploration (one-sample t-test). Scramble *n* = 7, shMbd2 *n* = 6.

Next, we asked whether hippocampal Mbd2 is casually involved in regulation of behaviors that are impaired in adult *Mbd2−/−* mice. We therefore examined whether Mbd2 down-regulation in adult mice affects some or all of the behavioral changes seen in *Mbd2−/−* mice which lacked the gene both during embryogenesis and post-natal development. To this end, we constructed several shRNA lentiviruses which targeted *Mbd2* mRNA (sh-*Mbd2*; Fig. S18A, B). The construct which was the most active in *Mbd2* depletion as tested in-vitro was infused in-vivo into the hippocampus of adult wild-type mice to knockdown Mbd2 (Fig. 5A, B). GFP-expressing-cells were found near the injection site and up to the dentate gyrus and stratum radiatum of CA3 (Fig. S18D–H). Following the infusion and recovery we tested the mice in the same behavioral tests that were found to be abnormal in *Mbd2−/−* mice. Down-regulation of Mbd2 did not affect locomotion (Fig. 5C) but increased self-grooming time and average grooming bout duration (Fig. 5D–F). Mbd2 down-regulation resulted in failure of treated mice to remember location of objects in the object-location-memory test. While control mice explore the object in the novel-location significantly more than the familiar location (*p* < 0.05, one-sample t-test), sh-*Mbd2*-treated mice did not explore the object in the novel location any longer than chance level (Fig. 5G). Interaction time in the social-interaction test was not affected by Mbd2 down-regulation (Fig. 5H). In the Dark-Light-Box test, sh-*Mbd2*-treated mice showed increased anxiety expressed as reduced time in the light compartment (Fig. 5I, J). Taken together, these results show that hippocampal Mbd2 knockdown in adult mice recapitulate most of the behavioral abnormalities found in *Mbd2−/−* mice but not the social interaction deficiency.

## Discussion

There is growing evidence for the crucial role of epigenetic mechanisms in neuropsychological disorders and CNS function. Here, we tested the role of Mbd2, a methylated-CpG binding protein, in gene-expression and brain function. Mbd2, like other MBD proteins, serves as a “reader” of the epigenome [3] translating DNA methylation marks into gene-expression regulation mechanisms. It has previously noted that loss of Mbd2 results in behavioral deficits such as impaired maternal care, poor nest building and mild memory impairment [23, 30]. Here we report that loss of Mbd2 results in cognitive, social and emotional deficits. Taken together these results imply a role for Mbd2 in normal brain function. Most of the phenotypic findings were replicated by hippocampus-specific down-regulation of Mbd2 in wild-type mice (increased self-grooming, impaired memory-retention in the object-location memory test, and anxiety-like behavior in the dark-light box) providing further evidence for the causal role of hippocampal Mbd2 in regulating behavioral phenotypes including long-term spatial memory and emotional control. Social interaction was not affected by *Mbd2* knockdown. It is possible that the level of inhibition of Mbd2 achieved by lentiviral knockdown was insufficient to affect social interaction. Alternatively, hippocampal Mbd2 might play a developmental role in social interaction behaviors and might not be required for maintenance of this behavior in adults. Another explanation is that Mbd2 is ubiquitously expressed in brain cells including glia (see Fig. S20), while our lentivirus targeted mostly neurons. Therefore, it is possible that some of the behavioral effects regulated by Mbd2 are based on its role in glial cells and they were not affected by neuronal Mbd2 knockdown. Since the effects of *Mbd2*-deficiency on behavior were broad and related to hippocampal function [34–38], we used genome-wide approaches to define the landscape of Mbd2 binding in the hippocampus and then determined how its deficiency affects the landscapes of DNA methylation and gene-expression using *Mbd2−/−* mice. Consistent with an important role in brain function as detected by the behavioral assays presented here, pathway analyses revealed a highly clustered and networked enrichment of genes relating to cognitive functions and brain development such as trans-synaptic signaling, synapse organization and behavior. Disruptions in these pathways may result in ASD (for review see: Gilbert and Man [63]).

Mbd2 regulates gene expression by binding to methylated CpGs in DNA [64]. MBD proteins are generally considered to have a repressive role on gene expression by interacting with chromatin modification inactivating complexes [65]. However, evidence suggests other MBD proteins are involved in gene expression in more than one way. For example, hypothalamic Mecp2 dysfunction led to robust changes in gene expression with 85% of the genes activated by Mecp2; a process possibly mediated by the interaction of the transcriptional-activator Creb1 with Mecp2 in binding gene-promoters and regulatory regions [66]. Similarly, Mecp2 promotes the expression of *Fopx3* in regulatory T-cells (Treg), a key regulator of Treg function, by collaborating with Creb1 [67]. Neuronal Mbd1 was also found to have a mixed effect on gene expression in the hippocampus with more than half of the genes downregulated in *mbd1−/−* mice, many of them associated with neurogenesis [21]. Mbd2 was previously shown to be involved in both suppression of promoters through recruitment of transcriptional repressors, and in activation of promoters in cancer cells and the hippocampus through recruitment of transcriptional-activators [17, 56–58] such as CBP and NGFIA [17, 58, 68] as well as by targeting DNA demethylation [69, 70] possibly through recruitment of oxygenases such as Tet2 [53]. In contrast, others have shown that MBD proteins binding to the DNA blocks Tet activity [71] and thus should inhibit demethylation. It is possible that MBD2 has a bimodal action on DNA methylation status that is dependent on its other partners blocking Tet and inhibiting demethylation in certain contexts while protecting hypomethylated sites in other contexts such as when Mbd2 is binding CBP or NGFIA [17, 58, 68]. Although Mbd2 shows low affinity to unmethylated DNA in vitro and does not contain the CXXC domain responsible for recognizing unmethylated CGs in Mbd1 [72], the situation in cells in the context of other protein complexes might be different. For example, Baubchek et al., have shown NuRD-complex-mediated tethering of MBD2 to a subset of unmethylated, active regulatory regions [5]. Certain transcription factors such as CBP and NGFIA [17, 58, 68] might also recruit Mbd2 to unmethylated promoters [73].

Consistent with these data we found evidence for the bimodal function of Mbd2 in the hippocampus. *Mbd2*-deficiency resulted in both gene activation and repression as well as hypermethylation and hypomethylation. Interestingly, the neuronal-related genes were mainly repressed and hypermethylated by *Mbd2*-deficiency, suggesting a mostly activating role for Mbd2 at neuronal-specific genes. This is supported by the observation that Mbd2 localizes to promoter and transcription start sites, and also by our findings of Mbd2 association with chromatin regions with bivalent histone marks, mostly in gene bodies, that were enriched for axon guidance and axonogenesis.

To further explore the role of Mbd2 in transcriptional regulation, we examined several Mbd2-bound promoters and enhancers tested the consequences of *Mbd2*-deficiency on transcription initiation. Most of the genes examined had reduced RNApolII(PS5) occupancy and increased H3K4me1 masking of the transcription-initiation region indicating reduced transcription-initiation upon *Mbd2*-deficiency.

Genetic and environmental factors are believed to be involved in the etiology of ASD and other neurodevelopmental disorders. DNA methylation has been proposed as an epigenetic interface that links environmental factors to genomic susceptibility in ASD [74–80] and neurodevelopment [81, 82]. MBD proteins as readers of DNA methylation are therefore potential mediators which translate the altered DNA methylation landscape into gene expression and ultimately behavioral changes. While the involvement of MBD2 in psychiatric disorders was previously suggested [13, 15, 83], our study is the first to provide a possible mechanism. Interestingly, the list of genes that we found here to be Mbd2-dependent was significantly enriched in ASD-associated genes according to brain transcriptomic and GWAS studies [26, 27]. Moreover, the behavioral phenotypes that we and others have characterized in Mbd2*−/−* mice resemble several of the phenotypes frequently observed in ASD individuals such as social avoidance, repetitive behavior, anxiety, and deficits in learning and memory. This is accompanied by morphological changes in the hippocampus similar to those previously reported in ASD individuals and ASD rodent models such as increased number of neurons [59, 62] and reduced thickness of hippocampal sub-regions [60, 61]. It is therefore possible that *Mbd2−/−* mice have impaired neuronal development which results in an increased number of presumably immature neuron but less mature neurons. This pattern has been described before in *Mbd1−/−* mice [12, 21].

Our findings further highlight the importance of MBD proteins in neuropsychological disorders. Our findings also provide evidence that Mbd2 is a regulator of neuronal gene expression, brain morphology, and behavior.

There are contradictory reports on whether Mbd2 binds either hydroxymethylated or unmethylated DNA (see [5] vs [84, 85]). Since we used bisulfite conversion, we cannot differentiate between methylated and hydroxymethylated CGs. We cannot, therefore, exclude the possibility that some of our observed effects of Mbd2 loss on increased DNA methylation are, at least partially, representing an increase in hydroxy-methylation and that some of methylated-CGs bound to Mbd2 are hydroxymethylated rather than methylated. It is also possible that some of the methylated CG mapped as binding Mbd2 are hydroxymethylated and these might have an opposite effect on gene expression particularly in enhancer regions which were previously shown to be enriched in 5-hydroxymethylcytosine [86]. Our sequencing findings were based on bulk analyses of the hippocampus and comparative analysis to data from human cortex. Future work that will use single-cell multi-omics and cell-type specific sequencing and ex-vivo neuronal culture work would refine our understating of the role of Mbd2 across different cell types, brain regions, and hippocampus subfields and its relation to ASD-like behavior. Also, while we did not find any effect of sex on behavior, it is possible that similar behavioral phenotypes are a result of different gene-expression regulatory networks between males and females. This can arise from X chromosome inactivation patterns for example. Therefore, we believe that future studies should include sex as a biological variable also for ex-vivo experiments even in the case of observed similarities in behavioral phenotypes.

## Supplementary information

Supplementary Material

Supplementary Table 1

Supplementary Table 2

Supplementary Table 3

Supplementary Table 4

Supplementary Table 5
