# Supplementary material for: Methyl-CpG binding domain 2 (Mbd2) is an epigenetic regulator of autism-risk genes and cognition
Source: Transl Psychiatry. 2023 Jul 13;13:259. doi: 10.1038/s41398-023-02561-9 (PMC10344909; doi:10.1038/s41398-023-02561-9)
Supplement: Supplementary file 1 — Supplementary Material [file 41398_2023_2561_MOESM1_ESM.docx]

**Supplemental Online Materials**

**Table of Contents:
Supplemental Methods and Materials
Figure S1-S21
References**

**Behavioral procedures**

All behavioral experiments were conducted on 10-12 weeks old mice. In cases of manual quantification of behaviors, the experimenter was blind to the experimental conditions.

*Open-Field and Self-Grooming.* Mice were placed in an open field (45×45 cm) apparatus with 30-cm-high walls. Their locomotor activity, exploration and self-grooming behaviors were measured for 5 minutes as we described before ^1,2^. The open-field test was also used as a habituation session for the object-recognition and object-location tests. Grooming was considered and scored when a mouse was performing the following repeated and stereotyped set of movements; elliptical bilateral or unilateral paw strokes, body licking and tail and genitals grooming and scratching as described before ^3,4^.

*Object-Location Memory (OLM).* Each mouse was placed for 5 min in the open field with two identical objects (A1 and A2) positioned in two adjacent corners. Exploration was defined as sniffing or touching the object with the nose, whiskers and/or forepaws. The exploratory preference for each object was calculated as the percent time (t) spent exploring that object relative to the total time spent exploring both objects [(tA2/(tA1+tA2) x100]. For the long-term memory (LTM) test conducted 24h after the training session, the same mouse was allowed to explore the field for 5 min in the presence of the same objects in the same settings with the exception that one of the objects was displaced to a novel location. Visual cues (adhesive tape) were placed on two adjacent walls as described before ^5^.

*Social Interaction Test.* The test was performed as described before ^6^. A Previous study did not find changes in social behavior in the three-chamber social approach test in Mbd2-/- mice ^7^. Therefore, we selected the social interaction test based on reports suggesting its increased sensitivity over the three-chamber social approach test ^6,8,9^. The duration of the test was 5 minutes. Sniffing, close following, and allo-grooming were defined as social interaction. No aggressive behaviors were found during the tests. The novel mice were wild-type mice from the same sex and same weight as the tested mouse.

*Spontaneous Alternations in Y Maze.* The Y maze was a standard 30 x 6 x 15 cm (Length X Width X Height) grey apparatus. Mice were allowed to freely explore the maze for 5 minutes. An entry to an arm was considered when all four paws were inside the arm. The measures included spontaneous alteration performance (i.e., a successful triad), alternate arm returns (AAR) and same arm returns (SAR). Total number of entries was assessed as well.

*Dark-Light-Box.* Anxiety-related behavior of the mice was tested in the Dark-Light Box consisting of two chambers connected by an open gate. Mice were placed near the gate in the light chamber facing the dark chamber. Latency to first entry to the light side, number of exits and total time in the aversive light compartment were recorded for 5 minutes.

We analyzed all the behavioral data for male and female mice and did not find any significant differences in behavior across sexes (two-way ANOVAs, p>0.05 for main effect of sex and for interactions in all cases, see also FigS1 and FigS2). We therefore grouped male and female hippocampi for all the following experiments with balanced numbers of male and females between groups.

**Lentivirus Production**

Three *Mbd2* shRNA plasmid were purchased from Dharmacon^TM^ (Lafayette, CO). The day before transfection, 1x 106 HEK293T cells were seeded in 10cm plate (DMEM medium supplemented with FBS and L-Glutamine, (Gibco, Ottawa, ON)). Next day, a 3 rd generation lentivirus was assembled by co-transfection of 4 vectors (5 µg each): pGIPZ vectors with one of three shRNA inserts or scramble sequence, a VSV-G envelope-expressing plasmid pMD2.G (Addgene plasmid #12259), and the packaging plasmids: pRSV-Rev (Addgene plasmid #12253) and pMDLg/pRRE (Addgene #12251). shRNAs sequences were: sh-*Mbd2*-1: 5’-AATTTGTTCTGTTACATCT-3’; sh-*Mbd2*-2: 5’- TTTCGGATCACTTCCTCCT-3’; sh-*Mbd2*-3: 5‘-TCTTCTGTAATTTACTAGG-3’; scrambled: 5’- GCCUUGGCAGCCUAGGCGA-3’. Lentivirus assembly was performed in the following manner: all four plasmids were mixed in 50 µl of OPTI-MEM (Invitrogen, Ottawa, ON). 45 µl of Fugene HD transfection reagent (Roche, Laval, QC) was diluted in 400 µl of OPTI- MEM. Plasmid mix was added to the diluted Fugene HD, mixed gently by pipetting and incubated for 30 min. at room temperature. Meanwhile, HEK293T cells were washed with PBSx1 and replaced with appropriate medium. The Fugene HD-DNA mix was added to the cell medium. After 48 hours of transfection of four plasmids, medium, containing virus was collected and passed through 0.45um filter.

***In-Vitro* Mbd2 Viral-mediated down-regulation**

In order to examine the ability of sh-*Mbd2* to down-regulate Mbd2 expression NIH3T3 cells (from ATCC) were infected with the three different sh-*Mbd2* lentiviruses. At 72 h after infection, cells were lysed, and the RNA extracted using Trizol according to the manufacturer's protocol. RNA was reverse-transcribed to cDNA using M-MuLV Reverse Transcriptase kit (NEB, Ipswich, MA) and cDNA was analyzed with QPCR.

**Stereotactic Surgery and Viral Infusion**

Eight-week-old mice were anesthetized with a Ketamine/Xylazine mixture and placed in a stereotaxic apparatus (Kopf Instruments, Tujunga, CA). Bilateral infusions of a shRNA-*Mbd2*-containing lentivirus (sh-*Mbd2*-1, or scramble control (n=9/group) were performed with a borosilicate glass capillary lowered to the hippocampus. Coordinates relative to bregma were: AP −1.9 mm, ML ±1.25 mm, and DV -1.75 to −2.0 mm; based on previous reports ^10^. Infusion was done with Auto-Nanoliter Injector (Drummond Nanoject II, Broomall, PA) at a rate of 38 nanoliter per infusion (lasts for 2 sec) followed by 20 seconds interval for a total of 2µl/hemisphere. The Glass capillary was remained in place for at least 2 minutes after the infusion to avoid reflux. Carprofen was injected post-surgery for at least 72h. Animals had 2 weeks recovery period before behavioral testing. Animals which had poor recovery or high levels of distress were euthanized according to the guidelines set by the Animal Care Committee of McGill University. We therefore performed the behavioral tests on 7 scramble-infused and 6 shMBD2-infused mice.

**Bioinformatic Analysis of the ChIP-Seq Data**

Read quality for all next-generation sequencing experiments was assessed using Fastqc (<http://www.bioinformatics.babraham.ac.uk/projects/fastqc/>) which confirmed high read quality and inconsequential levels of adapter contamination. Reads were aligned to the mouse reference genome (mm9 assembly) using Bowtie ^11^ with default parameters. Low quality alignments and alignments for read pairs with multiple possible genomic alignments were omitted. Duplicate read pairs were removed. Read count peaks corresponding to likely binding sites were identified for each sample by MACS2 ^12^ with callpeak –nolambda -q 0.05 parameters as described before ^13^, Mbd2-/- DNA reads were subtracted from the Mbd2 binding to control for nonspecific peaks. Peaks with false discovery rates less than 0.05 were selected as high-confidence binding sites. Sites were annotated with their genomic locations relative to nearby genes using HOMER with default parameters. De-novo motif discovery was done with HOMER with background adjusted to CpG context and the default cumulative binomial distribution test. We used the BedTools command “intersect intervals” with default parameters to identify Mbd2 peaks that overlap with different histone marks. Peaks are considered to overlap if there is at least 1bp of overlap between intervals. We defined bivalent loci as loci where Mbd2 peaks overlap with at least 1 peak of a repressive histone mark (H3K27me3 and H3K9me3) and at least 1 peak with active histone mark (H3K4me1, H3K36me3, H3K27ac, H4K20me1, H3K4me3).

**Q-ChIP**

Hippocampi from 5-6 mice per group as biological replicates for validation of ChIP-seq identified Mbd2 peaks were subjected to the same ChIP protocol used for the ChIP-Seq. To exclude the possibility of idiosyncrasy of the anti-Mbd2 antibody used in the ChIP-seq experiment, we used another antibody for this experiment (Imgenex, IMG147, Oakville ON) and IgG antibody (Santa Cruz, Mississauga, ON) served as an additional control. For RNApolII(SP5) and H3K4me1 ChIP experiments antibodies from Abcam were used (ab5408 and ab8895, respectively. Toronto, ON). Purified DNA was resuspended in 40ul elution buffer. For QPCR analysis, SYBR green qudantitative PCR was performed using the LightCycler® 480 system (Software 3.5, Roche Molecular Biochemicals). To determine the relative enrichment, the 2 -ΔΔCt method was used with normalization to IgG and input data.

**Quantitative polymerase chain reaction (qPCR)**

RNA was extracted from 5 Mbd2-/- and 9 wild-type mice as biological replicates for validation of RNA-seq, cDNA was prepared with random hexamer primers (Invitrogen, Ottawa, ON) and a reverse transcription kit (NEB, Ipswich, MA) according to the manufacturer protocol. *Actb* was used as the reference gene. SYBR green quantitative PCR (qRT-PCR) was performed using the LightCycler® 480 system (Software 3.5, Roche Molecular Biochemicals). To determine the relative concentration of mRNA expression, the 2 -ΔΔCt method was used.

**Analysis of Differentially Methylated Cytosines**

Sequences were aligned to the mm9 mouse reference genome using Bsmap v2.89 ^14^. Output data were strand-sorted, filtered, and deduplicated with Picard tools. Next, methylation levels and coverage levels were extracted with methratio.py command in Bsmap. Differential methylation cytosines were analyzed with methylKit R package ^15^ with FDR threshold of 0.05 and at least 10% difference in methylation levels. Differentially methylated positions were annotated with HOMER ^16^. De-novo motif discovery for differentially methylated promoters was done with RSAT ^17^. We created 200bp sequences, each centered at the differentially methylated CpG as input for this analysis and performed RSAT motif discovery followed by comparison to known motifs from JASPER Core Nonredundant Vertebrates database. All sequencing results were deposited in GEO .

**Targeted Sequencing of Bisulfite-Converted PCR Amplicons**

To validate the results obtained from the genome-wide capture bisulfite sequencing we used an independent group of littermate mice (5 wild-type, 3 *Mbd2-/-*). Hippocampal DNA was extracted as described above with AllPrep-DNA/RNA/miRNA-universal kit (Qiagen) and bisulfite-converted with EZ DNA Methylation-Gold Kit (Zymo Research). PrimerSuite software ^18^ (<http://primer-suite.com/>) was used to design PCR primers specific for bisulfite-converted DNA for genomic loci which we found to have Mbd2 binding peaks (see table S5 for chromosomal positions and gene names for the amplicons). PCR products were cleaned with a Qiagen kit, and libraries were prepared with NEBNext® Ultra™ II (NEB). Libraries were sequenced on Illumina MiSeq with pair-end 250bp reads. Analysis of amplicon DNA methylation was done as described above for the genome-wide capture bisulfite sequencing.

**Protein-Protein Network Analysis**

Differentially expressed genes which were identified also as ASD-associated genes, were analyzed for protein-protein association. We used STRING v10.5 ^19^ for this analysis with default settings (medium confidence threshold 0.4). **Tissue Processing**

Mice were anesthetized before trans-cardiac perfusion with ice-cold saline solution (pH 7.4). One hemisphere was dissected and stored at -80°C until further processing. The second hemisphere was post-fixed in 4% paraformaldehyde (PFA) in 0.1M phosphate buffer (PB, pH 7.4) for 16 hours then saturated and stored in a cryoprotectant solution (30% sucrose in 0.1% PB, pH 7.4) until sectioned into 40 micrometer thick sections using a freezing sledge microtome (SM 2000R, Leica, Wetzlar, Germany) at − 20°C.

**Immunofluorescence**

Briefly, four free floating sections per mouse (n=5 wild-type; n=7 *Mbd2-/-*) were blocked with 10% normal goat serum (NGS) in phosphate buffered saline-0.1% Tween 20 (PBS-T) for 1 hour at room temperature and then incubated with an Alexa Fluor 488-conjugated anti-NeuN antibody (1:200, MAB377X, Millipore, Etobicoke, ON) in 5% NGS in PBS-T overnight at 4°C. For Mbd2 immunostaining the following lentivirus infusion (n=7 scrambled control; n=6 sh-Mbd2), sections were incubated overnight at 4°C with anti-Mbd2 antibody (1:100, ab38646, Abcam, UK) in 5% NGS in PBS-T, followed by incubation with Alexa Fluor 594-conjugated secondary antibody for 2 hours at room temperature. Sections were then incubated in a Sudan black B staining solution (0.3% Sudan black in 70% EtOH) for 5 minutes. Finally, sections were washed 3 times for 5 minutes in PBS-T and 3 times for 5 minutes in PBS. Sections were then mounted, dried and coverslipped with Aqua Polymount (Polysciences Inc., Warrington, PA).

A competition assay was performed to determine the specificity of the MBD2 antibody to the MBD2 peptide. Anti-MBD2 antibody (1:100, Abcam, ab38646, lot#GR322847-1) was preincubated with the MBD2 peptide (NovusBio, NBP2-56565PEP, lot#000027821) at 1 and 5 times the gram-amount of MBD2 antibody and the absorption solution was incubated for 1 hour at room temperature before performing the immunostaining as described above. Images were acquired using a confocal microscope Leica TCS SP8 (Leica, Canada) using a 40x oil objective and the LAS X Software (Leica, Canada). **Image Analysis**

To quantify neuron-number, three regions centered over the appropriate cellular layer CA1, CA2, CA3 were imaged on each of the four sections per animal using an Axio Imager M2 microscope and ZenPro software (Carl Zeiss Canada). Images were saved with 16-bit depth and were processed and analyzed using NIH ImageJ software (National Institutes of Health, USA). Data from all slices from each mouse was summarized and used as a single biological replicate.

To demonstrate virus targeting and Mbd2 downregulation, 15μm tiled z-stacks of the dorsal hippocampus were acquired with a 20x objective using an LSM710 Confocal Laser Scanning Microscope (Carl Zeiss AG, Germany). Images were then converted to maximum intensity projections using Zen 202 SP5 Black (Carl Zeiss AG, Germany). Acquisition and display settings were held constant for comparative images. Brightness and contrast adjustments were applied to whole images and were held constant across groups.

**Western Blot of Hippocampus tissue**

Hippocampi were harvested, lysed in RIPA buffer, loaded into 12% SDS-PAGE gel and transferred to nitrocellulose membrane. Membranes were then probed with antibodies against Drd2 (1:200, Bioss, Woburn, MA), Pogz (1:1000, Bethyl, Montgomery, TX) and Zbtb16 (1:250, Santa-Cruz, Mississauga, ON) Beta-actin was used as a reference protein for Pogz and Zbtb16 (1:5000, Sigma-Aldrich, Oakville, ON). For Drd2 total protein density measured by Ponceau Red was used for normalization since Drd2 and beta-actin bands were at about the same size. Blots were scanned by Amersham Imager 600 imaging system (GE Healthcare, Canada). Images of uncropped blots are in the supplements (Fig S19). Possible outliers were visible in the data; therefore, we applied the Iglewicz and Hoaglin's test for outliers under the strict criterion of z-score ≥|3.5|, which resulted in exclusion of no more than a single data point from each experiment.

**Statistical Analysis**

Data are expressed as mean ±SEM unless otherwise stated. Behavioral data were analyzed by two-way ANOVAs with genotype (wild-type/*Mbd2-/-*) and sex as the main factors, followed by HSD Tukey post-hoc tests. Comparisons between two groups were done with two-tailed t-test. Bisulfite-Converted PCR-amplicons results for validation DNA methylation were analyzed as one-tailed tests, based on the a-priori assumption to find differences in the same direction observed by the preceding capture bisulfite sequencing experiments. Data analysis from qpcr, immunohistochemistry and immunoblots was done by one-sample two-tailed t-test, comparing with a reference value of wild-type samples. In addition to a between-group, a one-sample t-test was applied on data from the object-location memory test to assess if novel-location-memory performance is above chance (50%) within each group. For correlation analysis between DNA methylation and gene expression, the Pearson’s correlation coefficient was calculated (cutoffs: differential methylation |≥25%| and log2 fold-change gene-expression |≥ 0.27|). Other correlations were calculated with Pearson’s R coefficient or Spearman’s Rho coefficient as detailed in the results section. Other statistical analyses of bioinformatic data are detailed in the results section. Heatmaps were generated with Morpheus (<https://software.broadinstitute.org/morpheus/> ; Broad Institute). GO annotations, pathway enrichment analysis and gene-networks were analyzed with Metascape (<http://metascape.org>) ^20^.

**Supplementary figures legend**


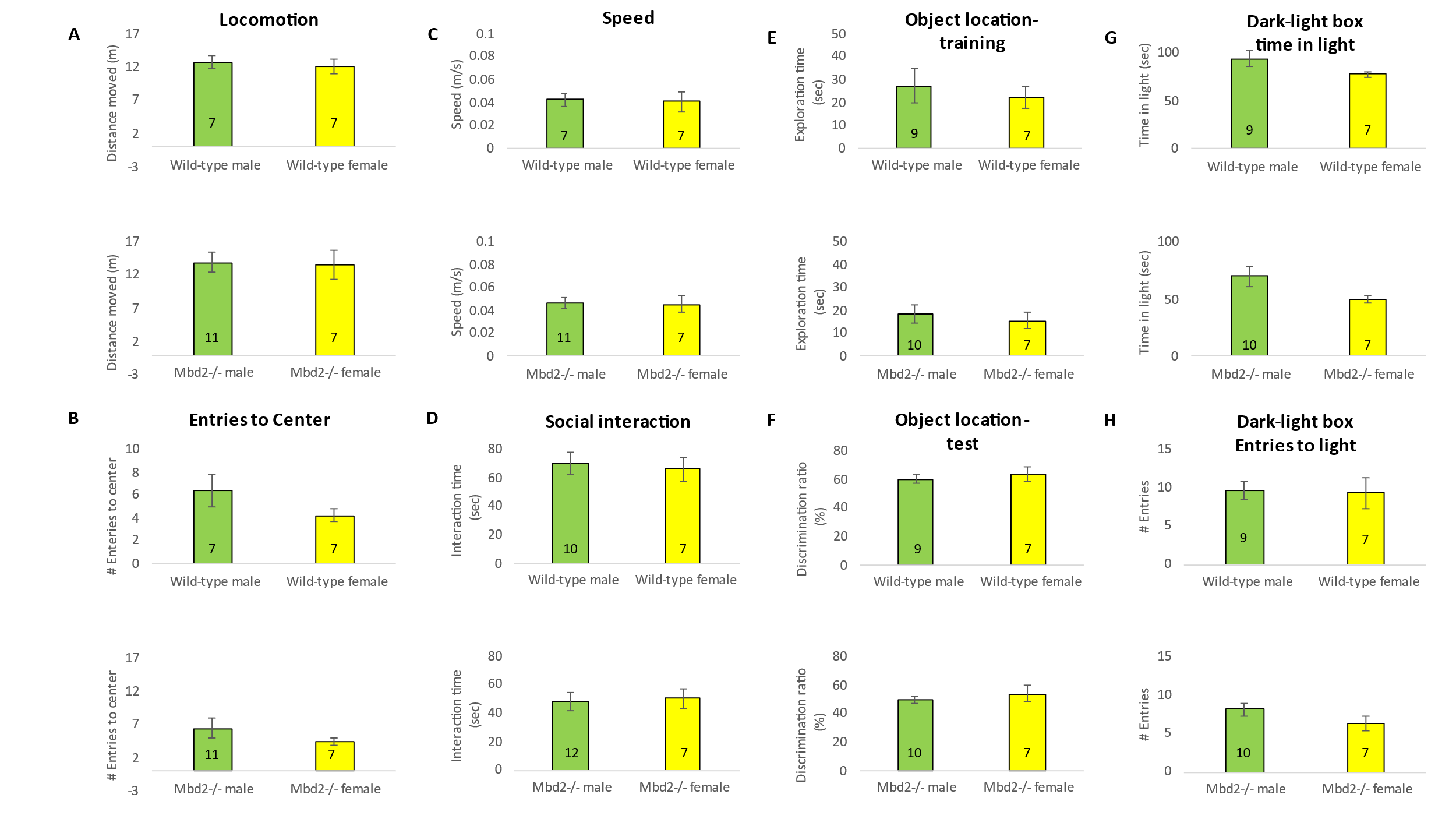


**Supplementary Figure 1.** A. Behavioral analyses of male and female *Mbd2-/-* and control mice A. Locomotion in Open-field box was assessed for 5 min. B. Number of entries to center in the open-field test. C. Average speed during the open-field test. D. Social interaction, mice were introduced to a novel mouse for 5 min and interaction time was recorded. E. Exploration time during object-location memory training and F. Discrimination ratio in object-location memory test. G. Time in the light compartment of the Dark-Light Box and H. number of entries to the light side. Numbers within bars represent sample size.


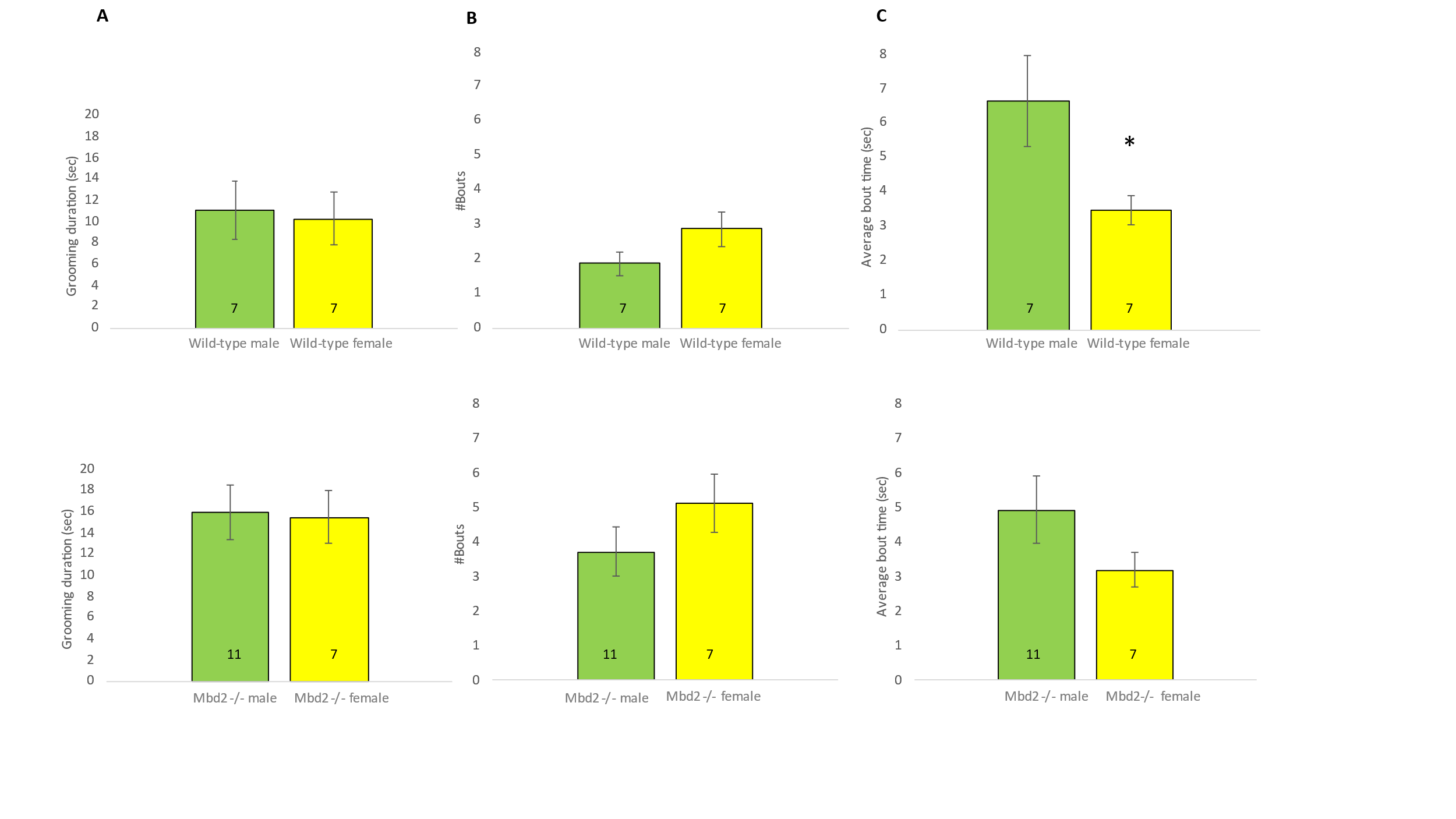
**Supplementary Figure 2.** Analysis of self-grooming behaviors: grooming duration, number of grooming bouts and average bout duration in male and female wild-type and Mbd2-/- mice. A two-way ANOVA found a main effect of sex F(1,28)=7.09, p= 0.0128 on average grooming bout duration. Subsequent independent t-tests found significantly shorter grooming bout duration in wild-type females compared to males.


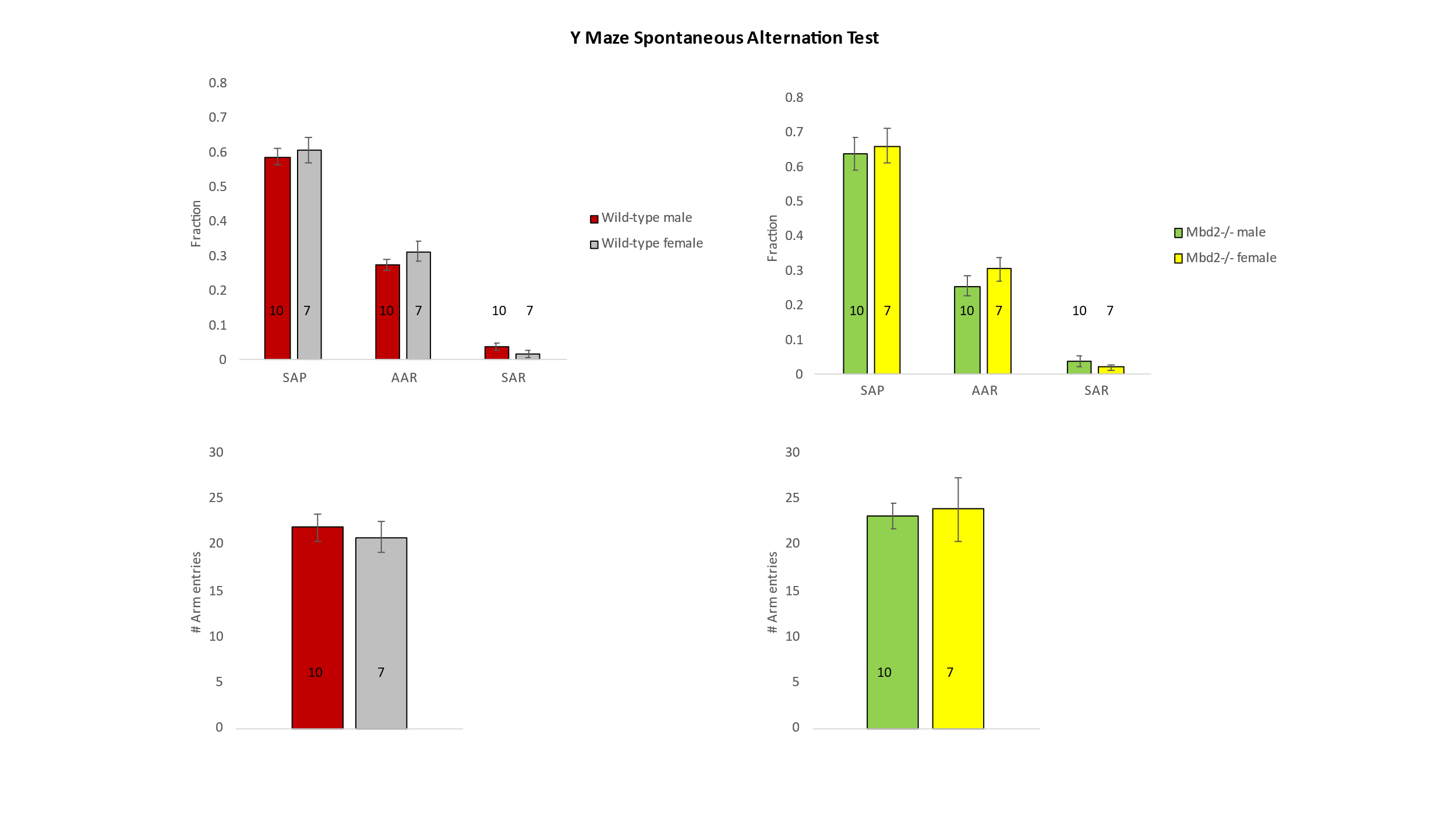


**Supplementary Figure 3.** Top: Y-maze spontaneous alteration. SAP-Spontaneous Alteration Performance, AAR-Alternate Arm Return, SAR-Same Arm Return, B. Bottom: Exploration is expressed as number of arm entries. Numbers within bars represent sample size.


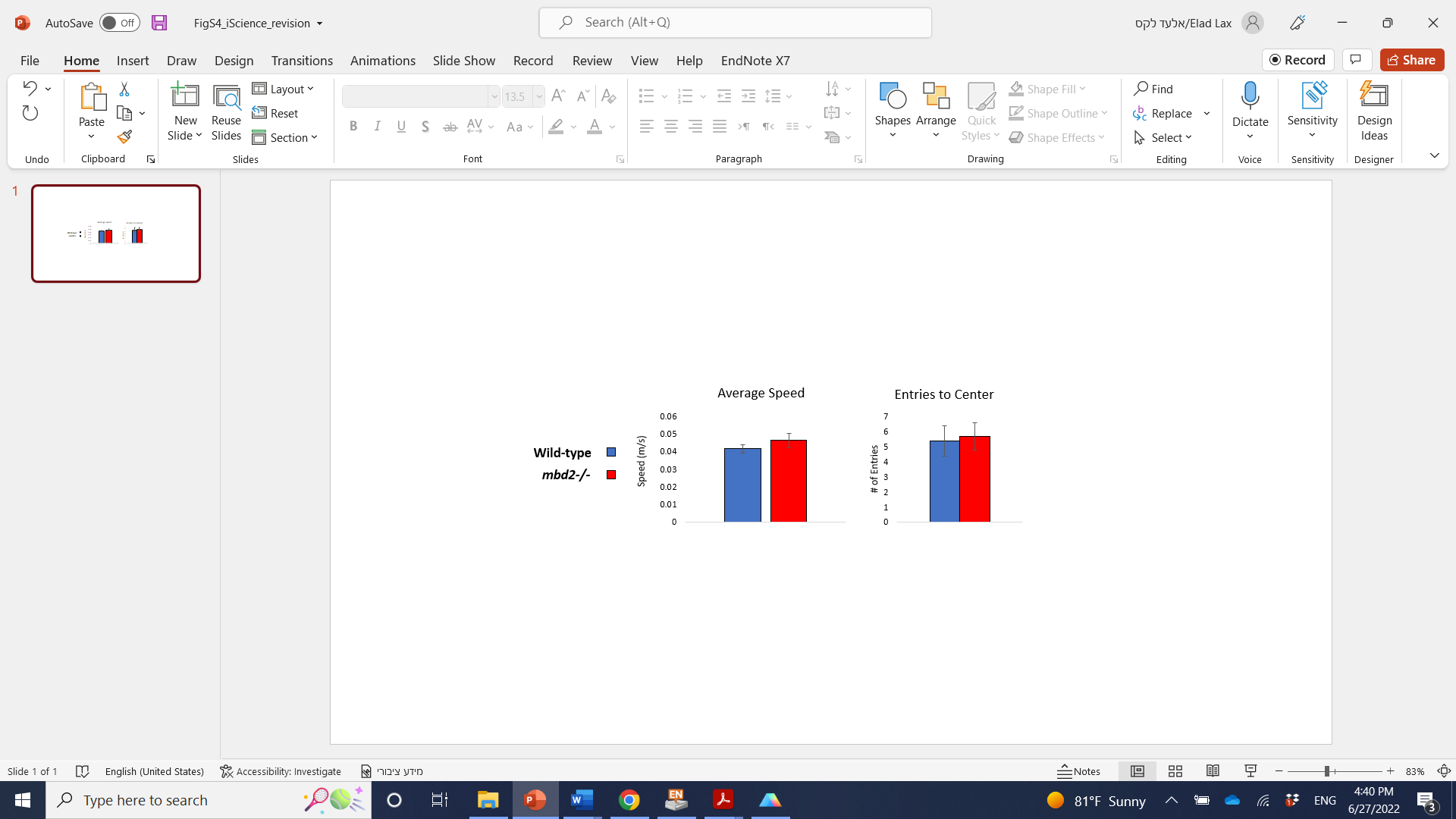


**Supplementary Figure 4.** Average speed (left), entries to center (right) in the open-field box test. p>0.05 in all cases.


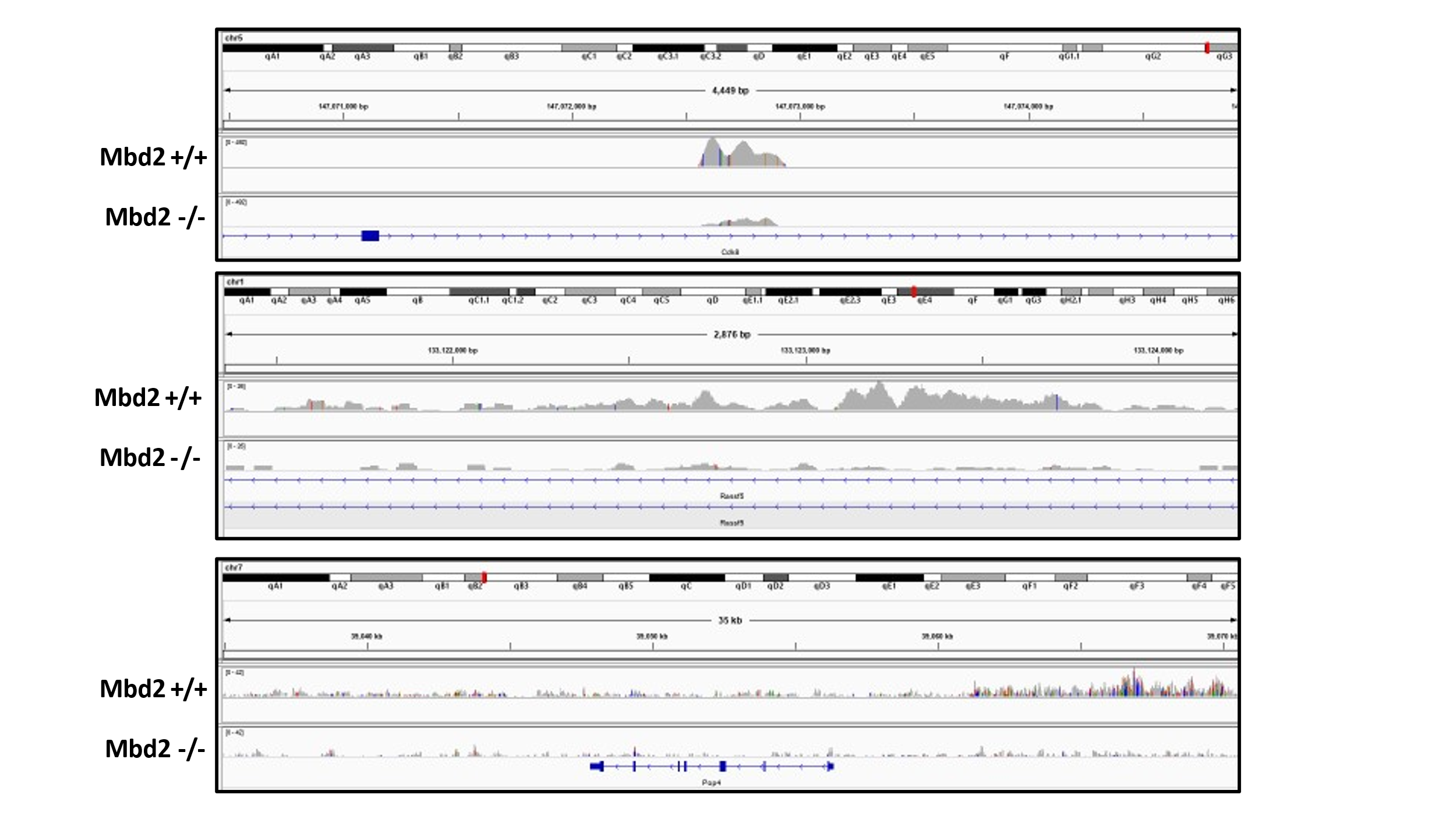


**Supplementary Figure 5.** Captures from genome browser showing Mbd2 ChIP-seq peaks from wild-type and Mbd2-/- (as background) mice annotated to *Cdk8* (top) and *Rassf5* (middle) gene bodies, and upstream of *Pop4* (bottom).


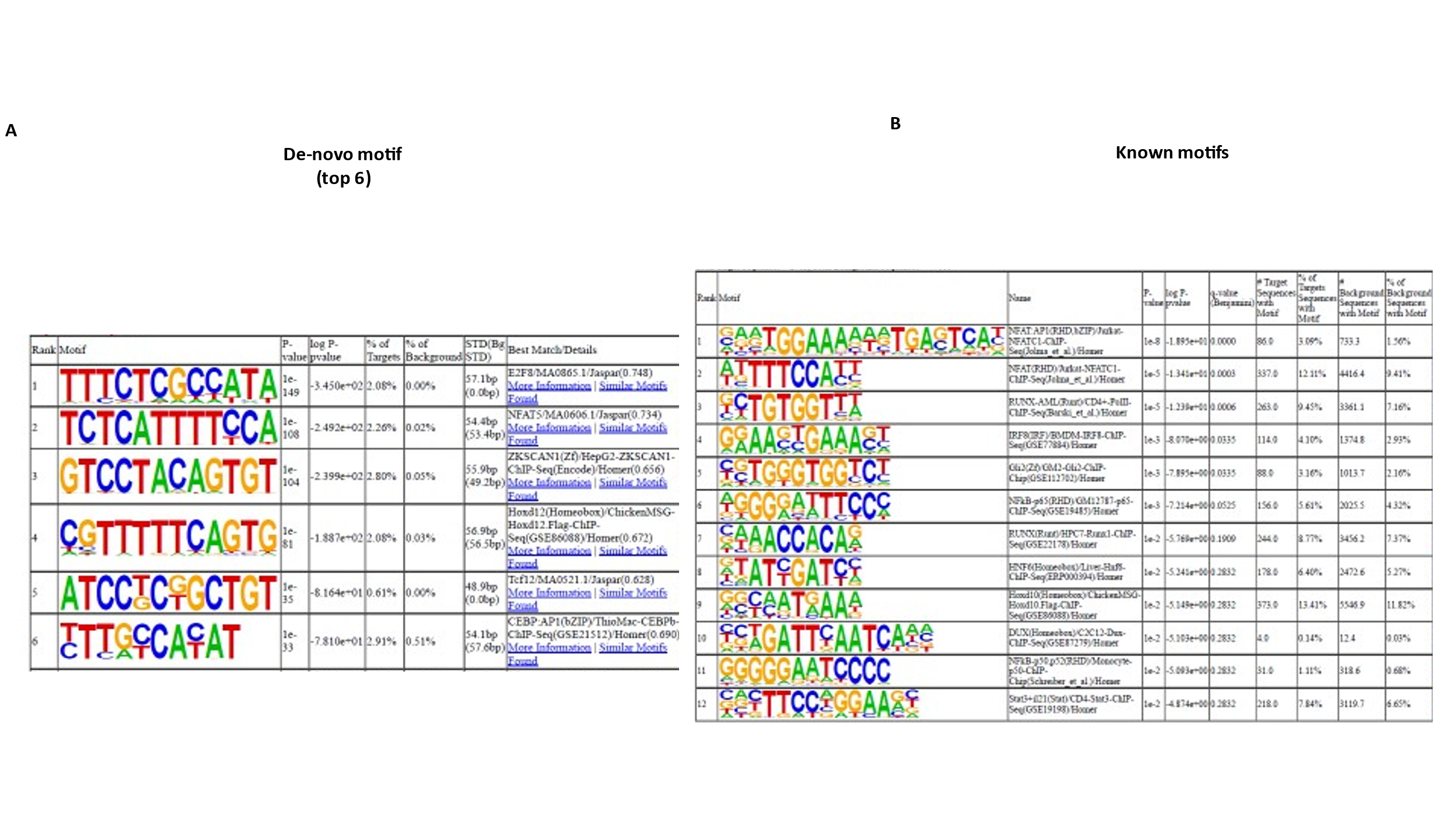
**Supplementary Figure 6.** A. De-novo motif discovery sequences found in HOMER analysis for Mbd2 ChIP-seq peaks. B. Known motif discovery sequences found in HOMER analysis for Mbd2 ChIP-seq peaks.


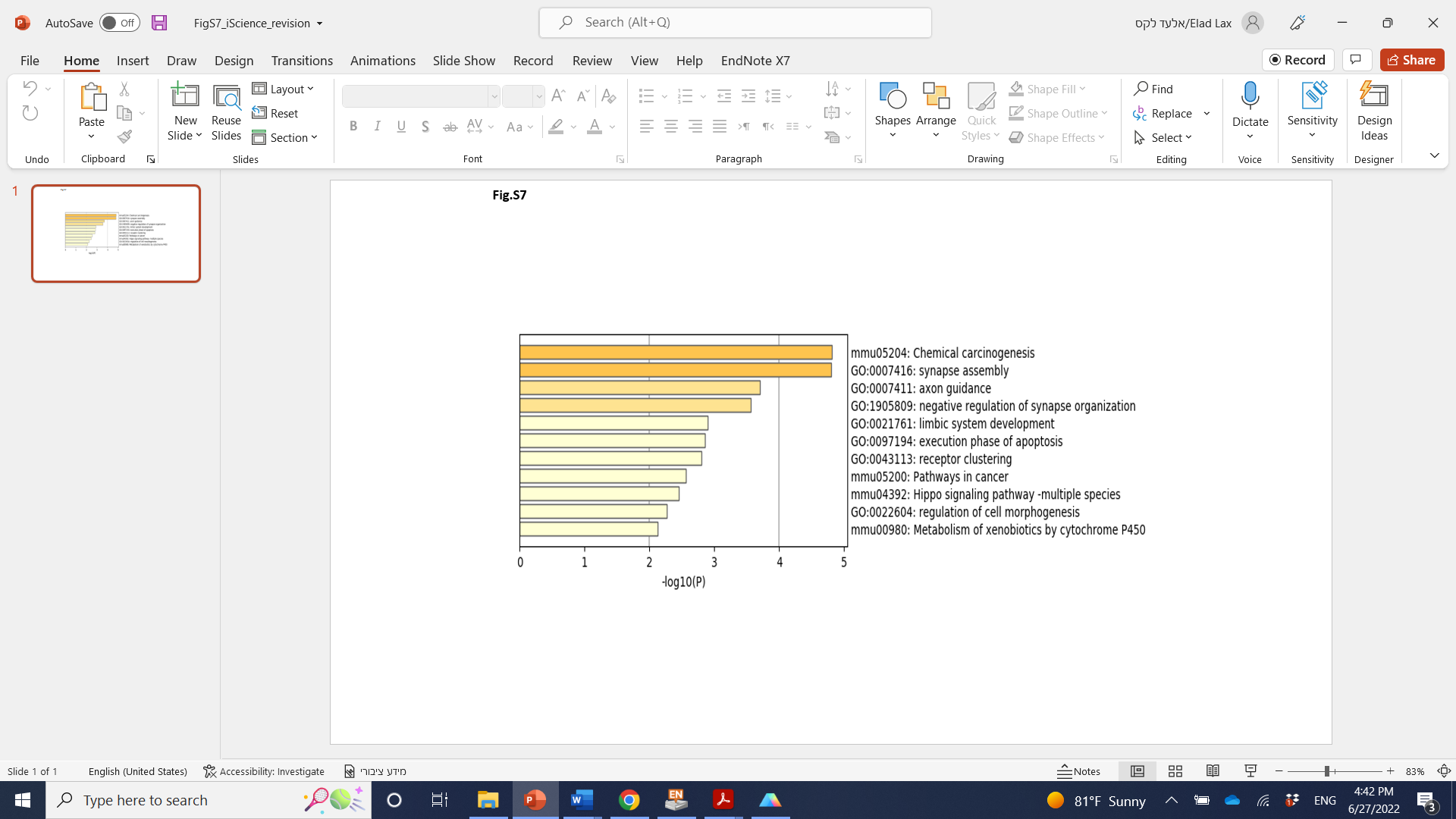
 **Supplementary Figure 7.** A. GO-Pathway analysis enrichment of Mbd2-bound genes. B. Gene-network analysis of Mbd2 binding peaks.


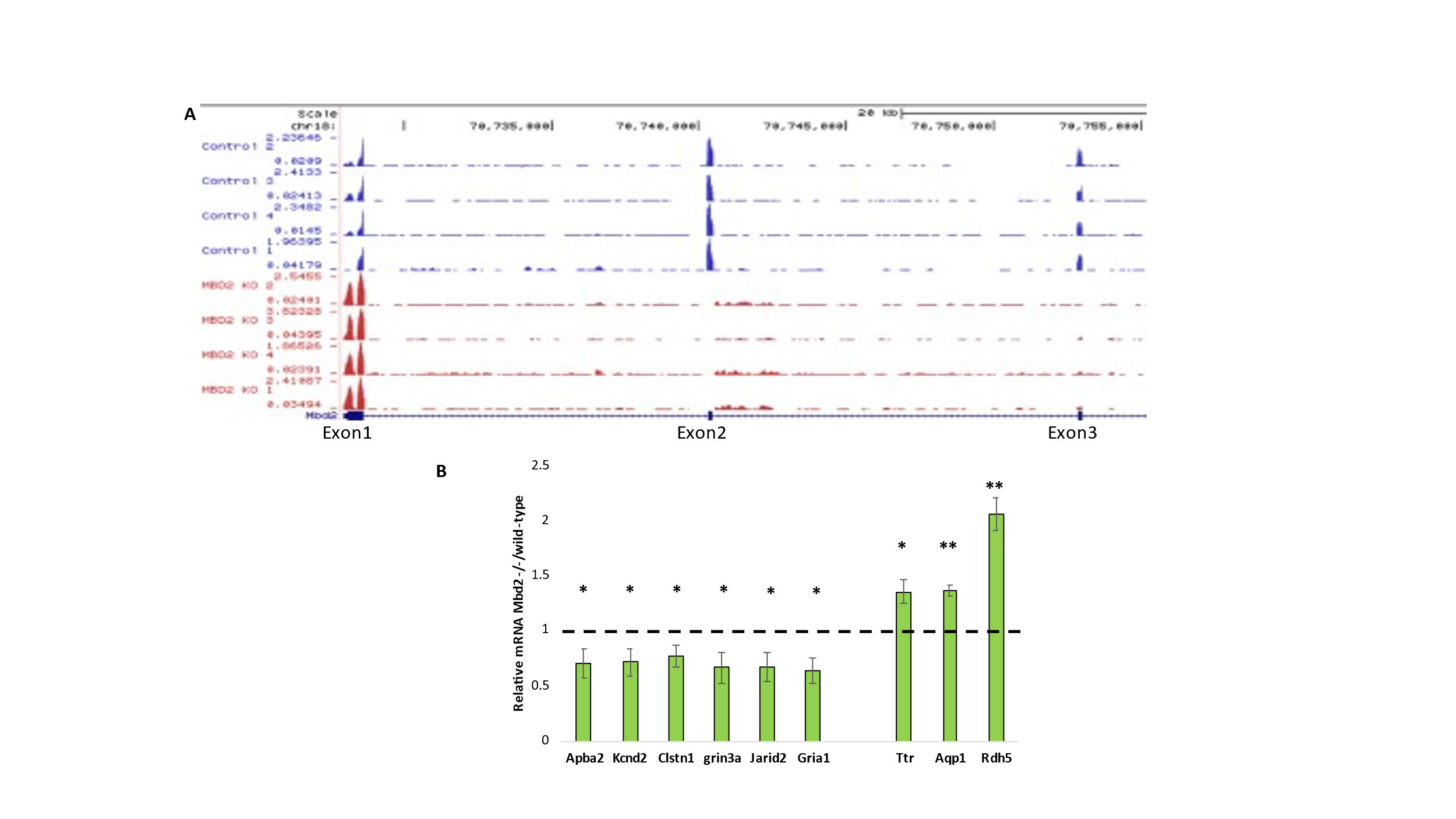
**Supplementary Figure 8.** A. A capture from genome browser showing RNA-seq read alignments for the first 3 exons of the *Mbd2* gene. *Mbd2-/-* mice show expression of the first exon only, as expected by the design of this mouse line ^21^. **B.** QPCR validation for down-regulated genes (*Apba2, Kcnd2,* *Clstn1, Gria3a, Jarid2, Gria1*) and up-regulated genes (*Ttr*, *Aqp1*, *Rdh5*) shows change in gene-expression in *Mbd2-/-* mice in agreement with the RNA-Seq data (n=9 wild-type, n=5 *Mbd2-/-*; *p<0.05, **p<0.01 t-test).


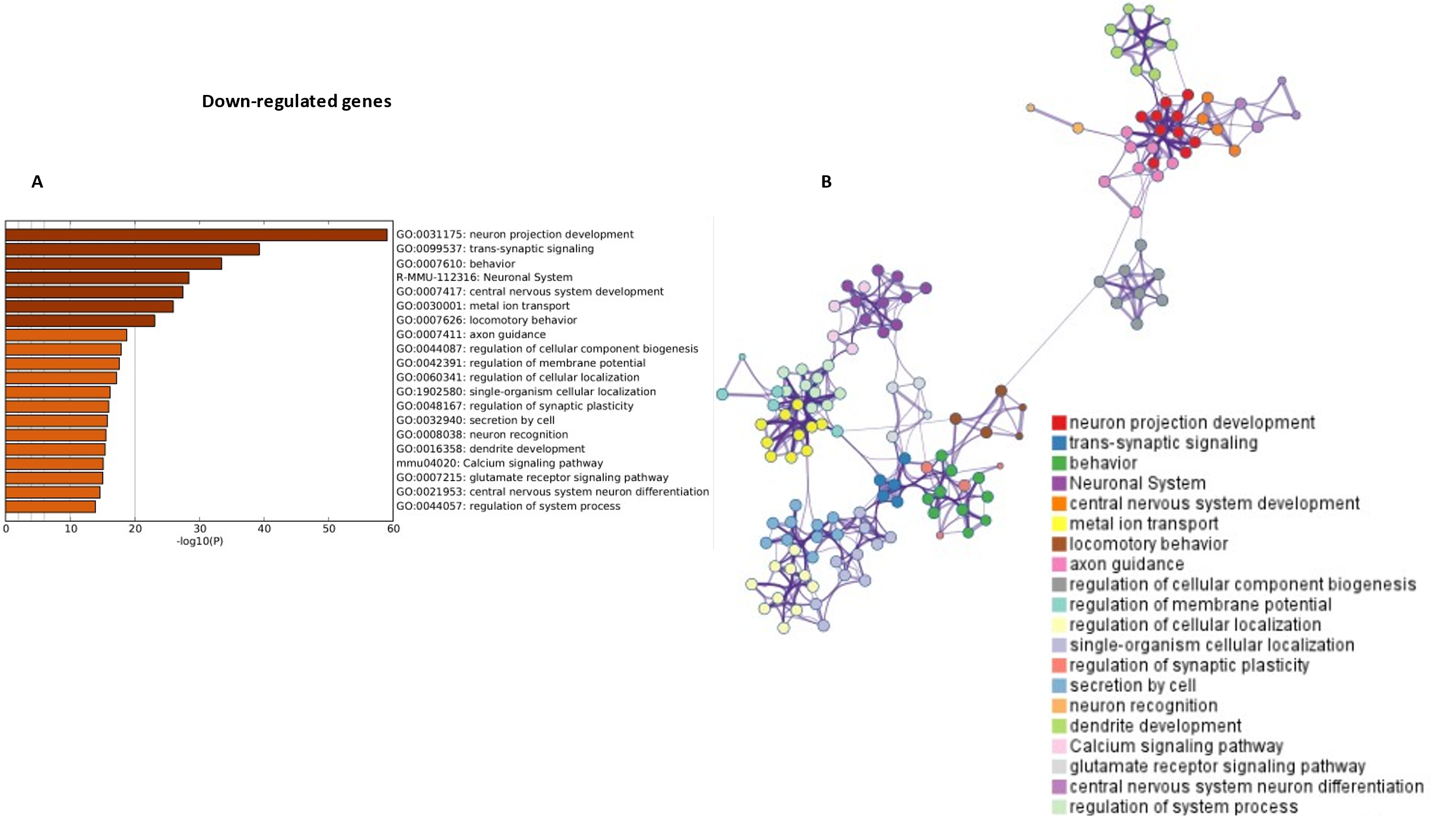
**Supplementary Figure 9.** A. GO-Pathway analysis enrichment of *Mbd2-/-* down-regulated genes. B. Gene-network analysis by Metascape of *Mbd2-/-* down-regulated genes.


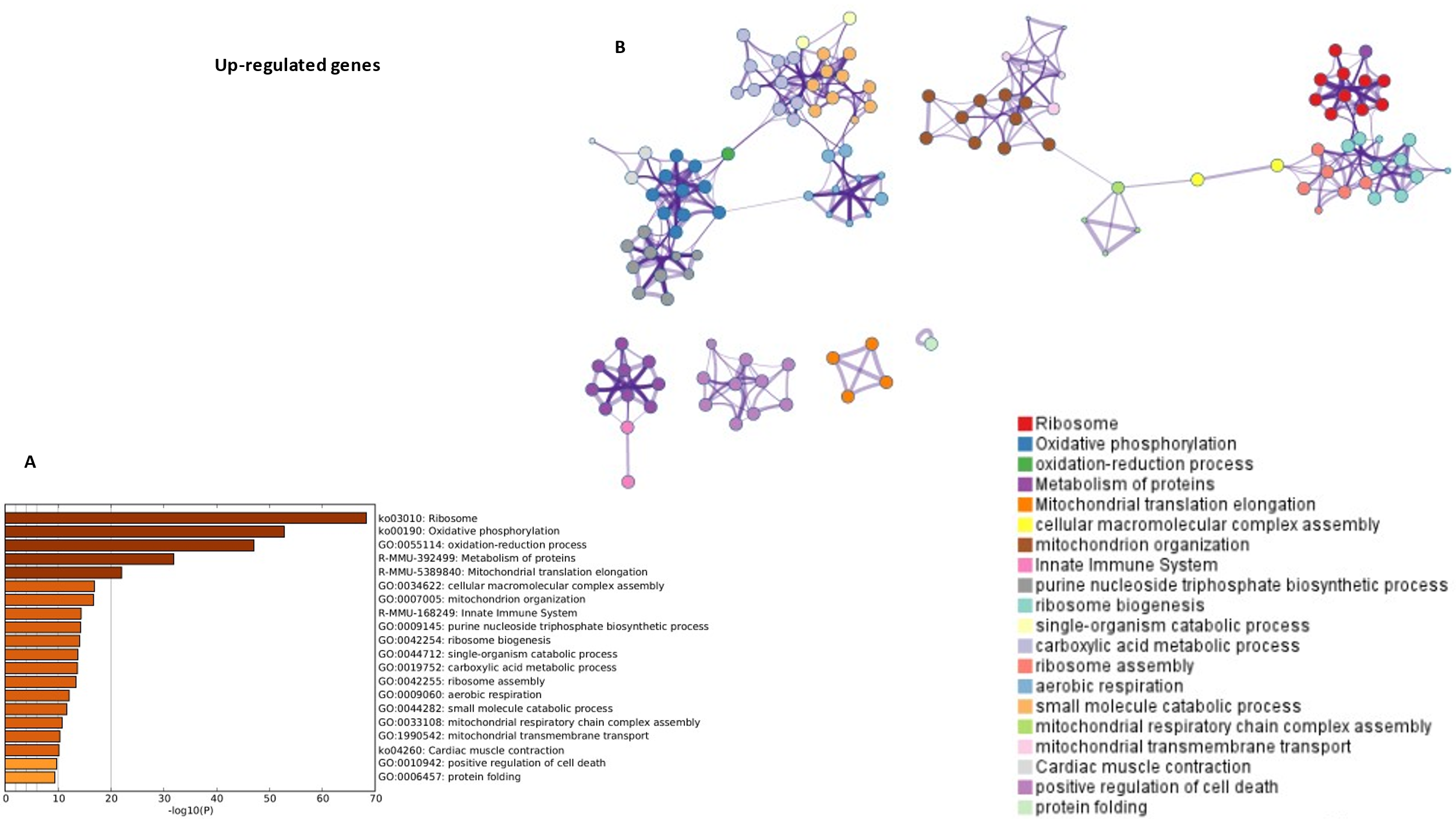


**Supplementary Figure 10.** A. GO-Pathway analysis enrichment of *Mbd2-/-* up-regulated genes. B. Gene-network analysis by Metascape of *Mbd2-/-* up-regulated genes.


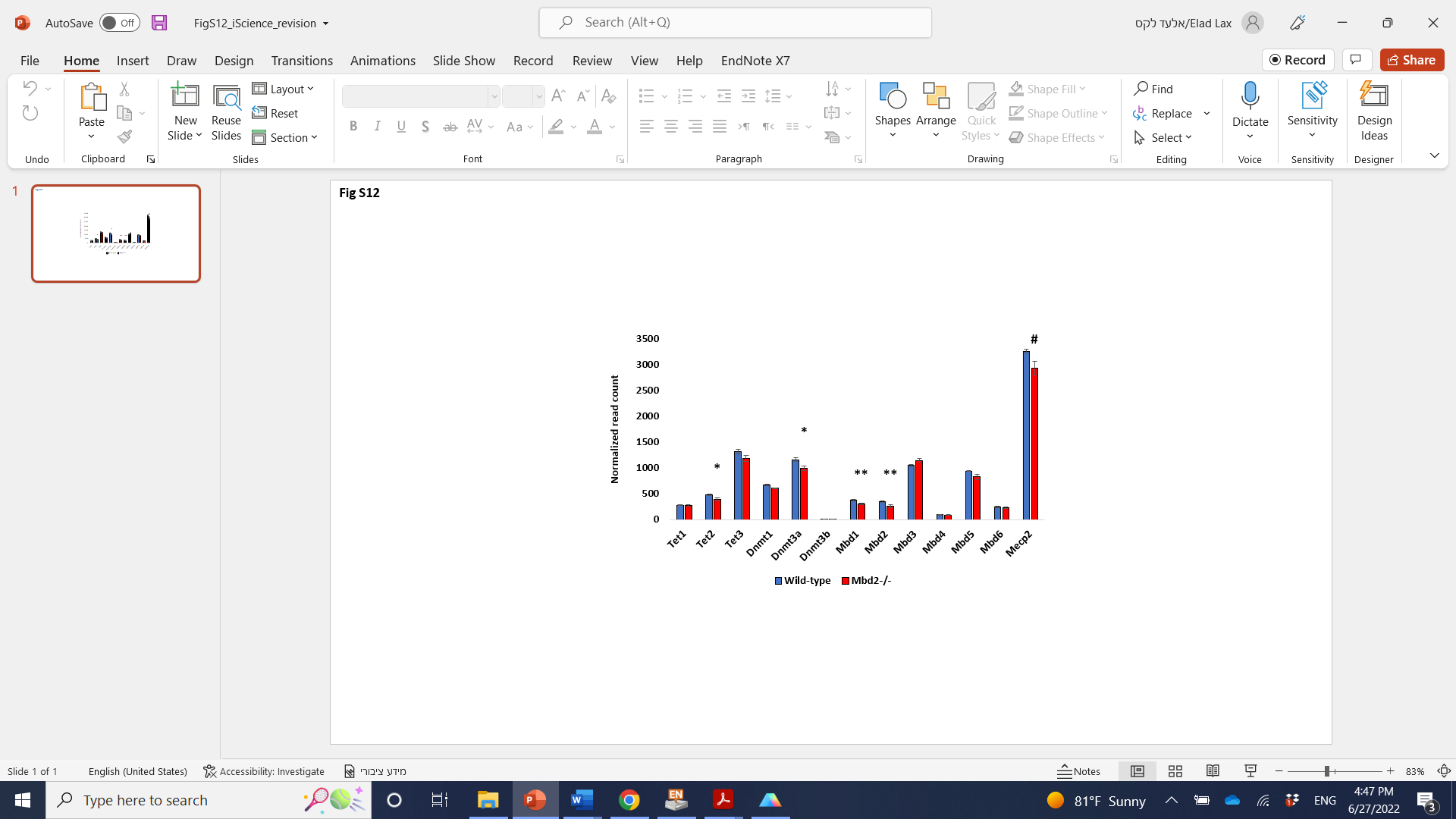


**Supplementary Figure 11.** Effect of *Mbd2-/-* on gene expression of epigenetic readers and modifiers. *FDR-corrected p<0.05, **FDR-corrected p<0.01, ^#^FDR-corrected p<0.1. Note: Mbd2-aligned reads in *Mbd2-/-* mice are from the intact exon 1 (see also Fig S6A).


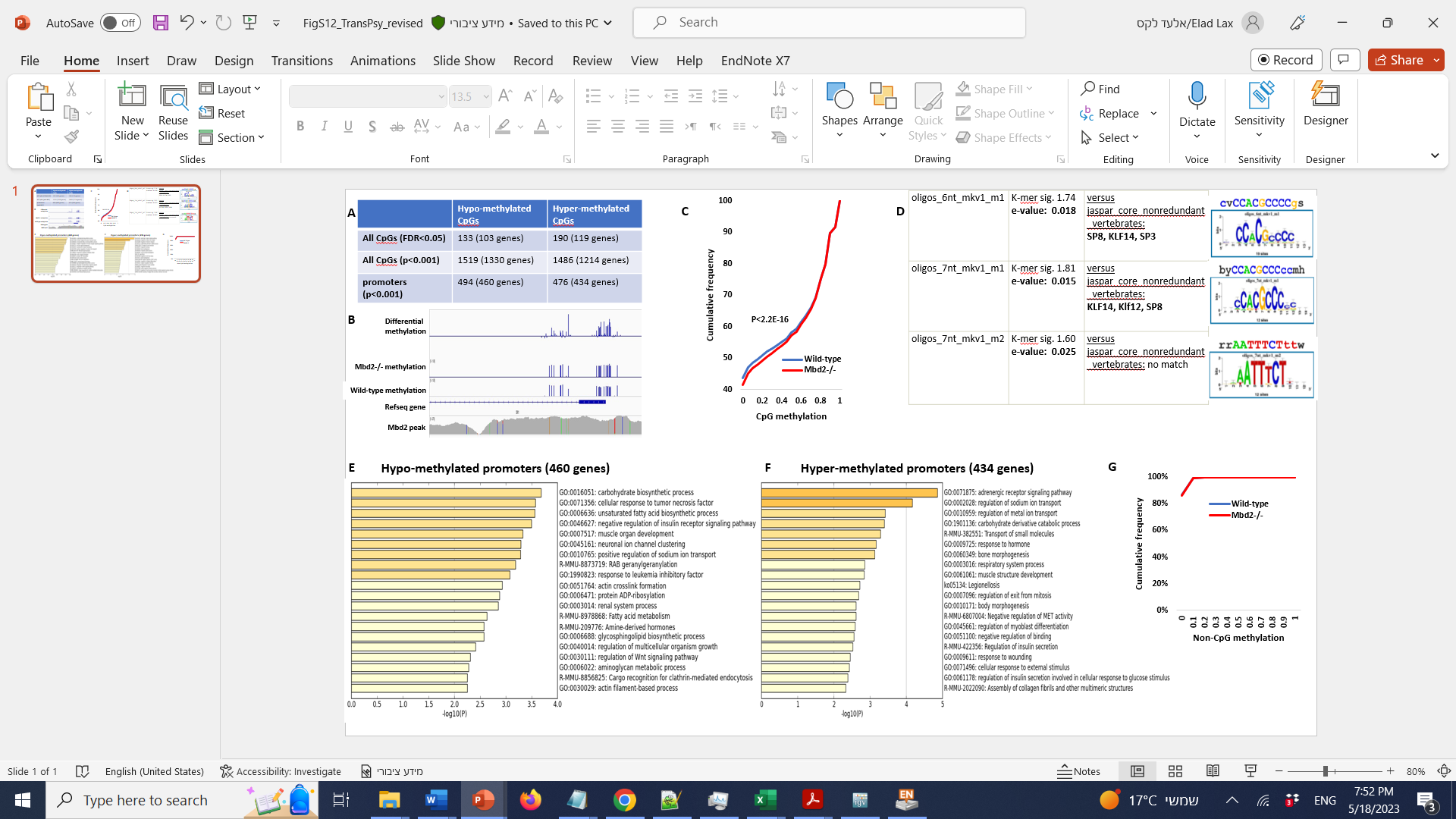


**Supplementary Figure 12.** A. A table summarizing the number of differentially methylated CpGs and genes under the different analytical conditions. B. A capture from IGV genomic browser depicting Mbd2 binding peak around Sfi1 transcription start site (TSS) with methylation levels and differential-methylation between wild-type and *Mbd2-/-* mice hippocampus. C. Cumulative distribution of methylation of regulatory DNA regions genome-wide demonstrating hyper-methylation in *mbd2*-/- hippocampus (K-S test). D. Significant motifs discovered for promoter CpG with positive correlation between DNA methylation and gene-expression are depicted. No significant motifs were found for CpGs with negative correlation between DNA methylation and gene-expression. E-F. Pathway analysis of hypo- (E) and hyper- (F) methylated gene promoters. G. Cumulative distribution of non-CpG methylation located at Mbd2 binding peaks.


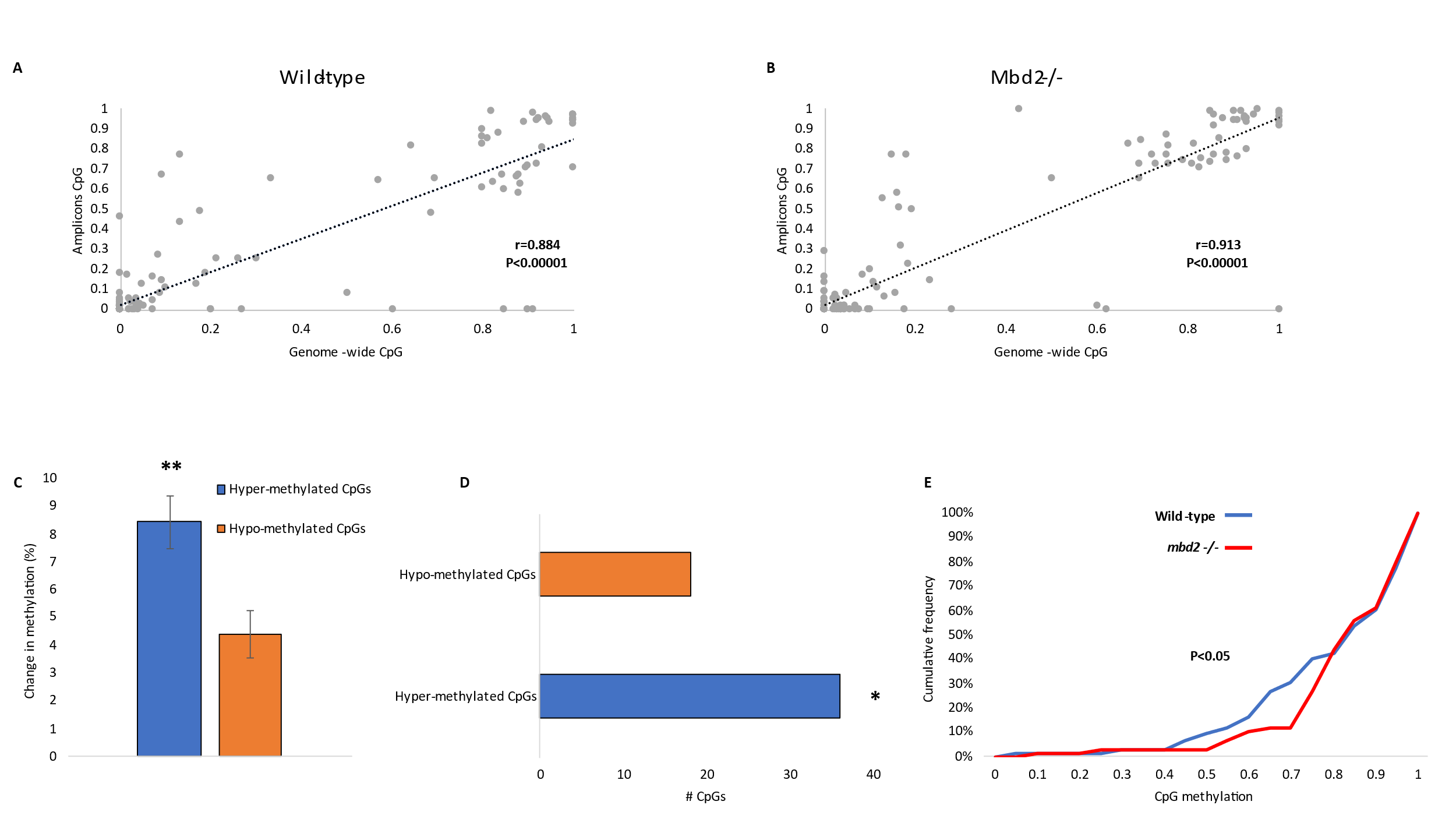
**Supplementary Figure 13.** A. Correlation analysis of CpG methylation levels in capture-array genome-wide bisulfite-sequencing and amplicons bisulfite-sequencing for wild-type mice. B. Correlation analysis of CpG methylation levels in capture-array genome-wide bisulfite-sequencing and amplicons bisulfite-sequencing for Mbd2-/- mice. C. Average change in methylation levels across all CpGs (with at least 1% change in methylation) show larger change in methylation between Mbd2-/- and wild-type mice in CpGs that became hyper-methylated (n=114) than in CpGs that became hypo-methylated (n=80) (t-test, **p<0.01) D. More CpGs with at least 1% change in methylation between mbd2-/- and wild-type mice are hyper-methylated than hypo-methylated (binomial test, *p<0.05). E. A cumulative distribution of methylation levels within Mbd2 peak regions in the validation study (K-S test).


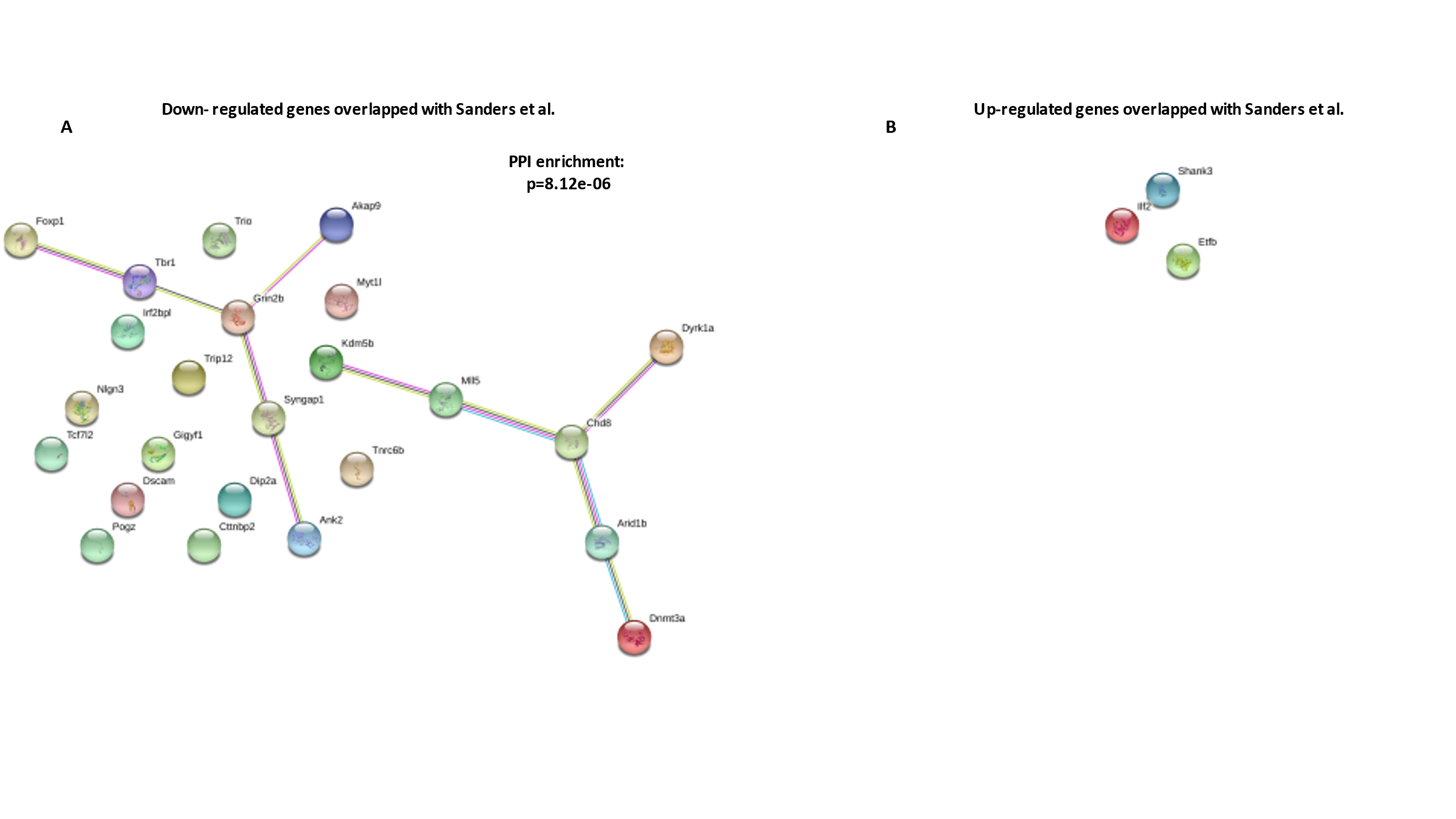
**Supplementary Figure 14.** A-B. Protein-Protein Interaction Networks for down- (A) and up- (B) regulated genes overlapped with ASD risk genes (Sanders et al^22^). PPI=Protein-Protein Interaction.


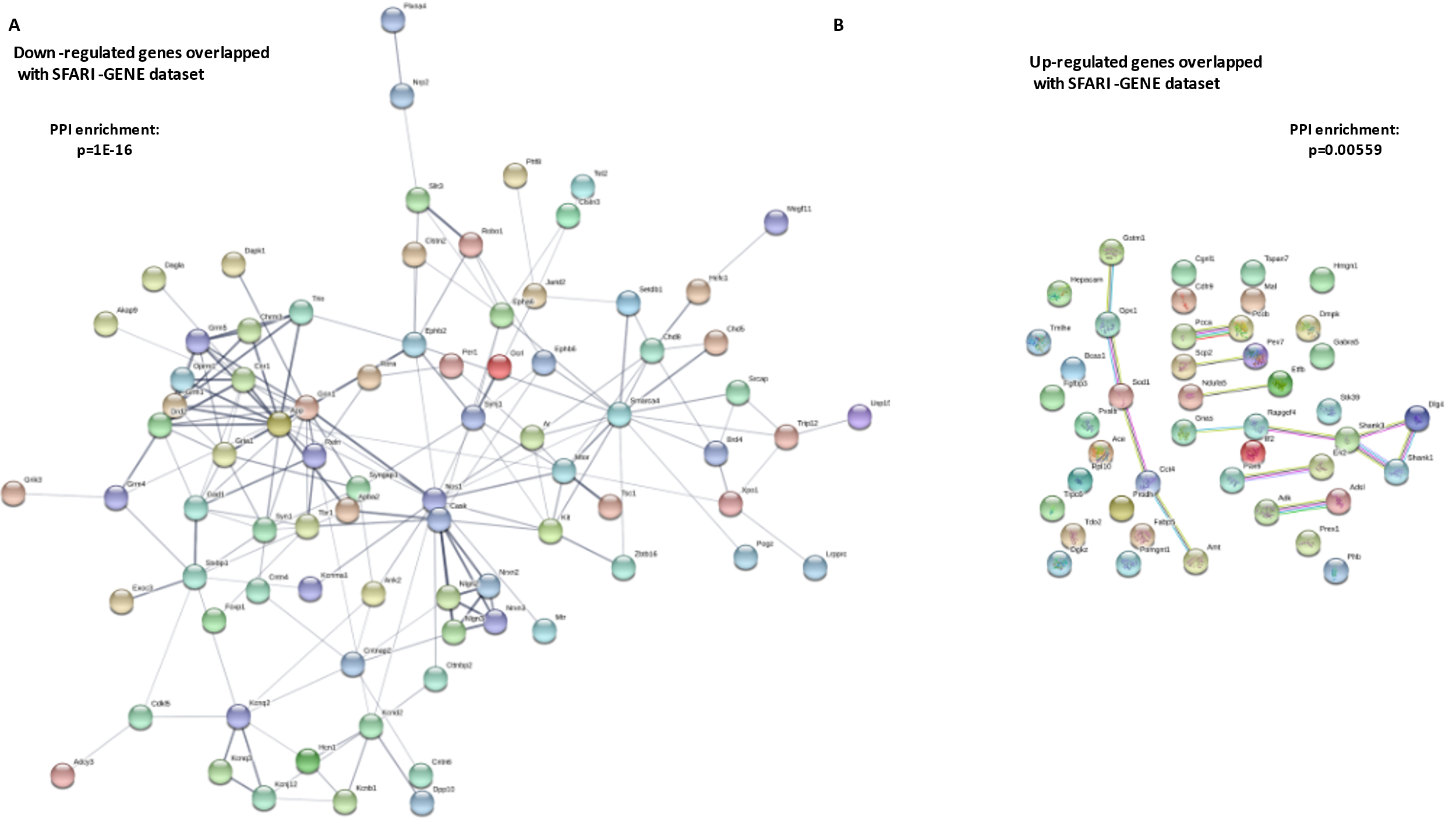


**Supplementary Figure 15.** A-B. Protein-Protein Interaction Networks for down- (A) and up- (B) regulated genes in *Mbd2-/-* overlapped with SFARI-GENE dataset. PPI=Protein-Protein Interaction. Disconnected nodes are omitted from (A) for clarity.


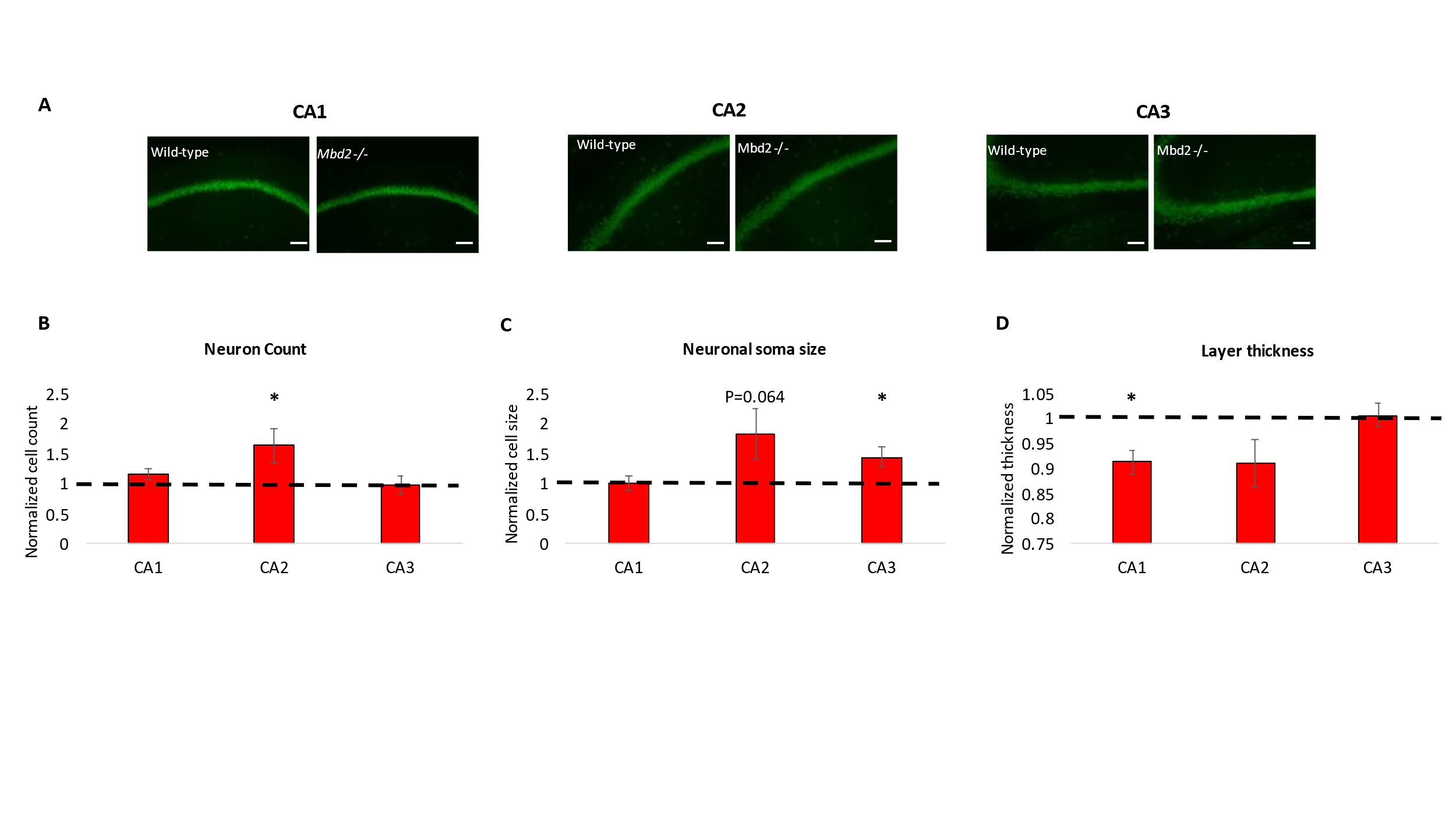
**Supplementary Figure 16.** A. Representative immunofluorescence images of the hippocampus (CA1-CA3 layers) in wild-type and *Mbd2*-/- mice showing neuronal nuclei immunoreactivity (NeuN, green immunoreactivity). Scale bar: 100μm. B. Quantification of neuronal cell count in hippocampal layers. C. Quantification of average neuronal soma size in hippocampal layers.
D. Average thickness of hippocampal layers.


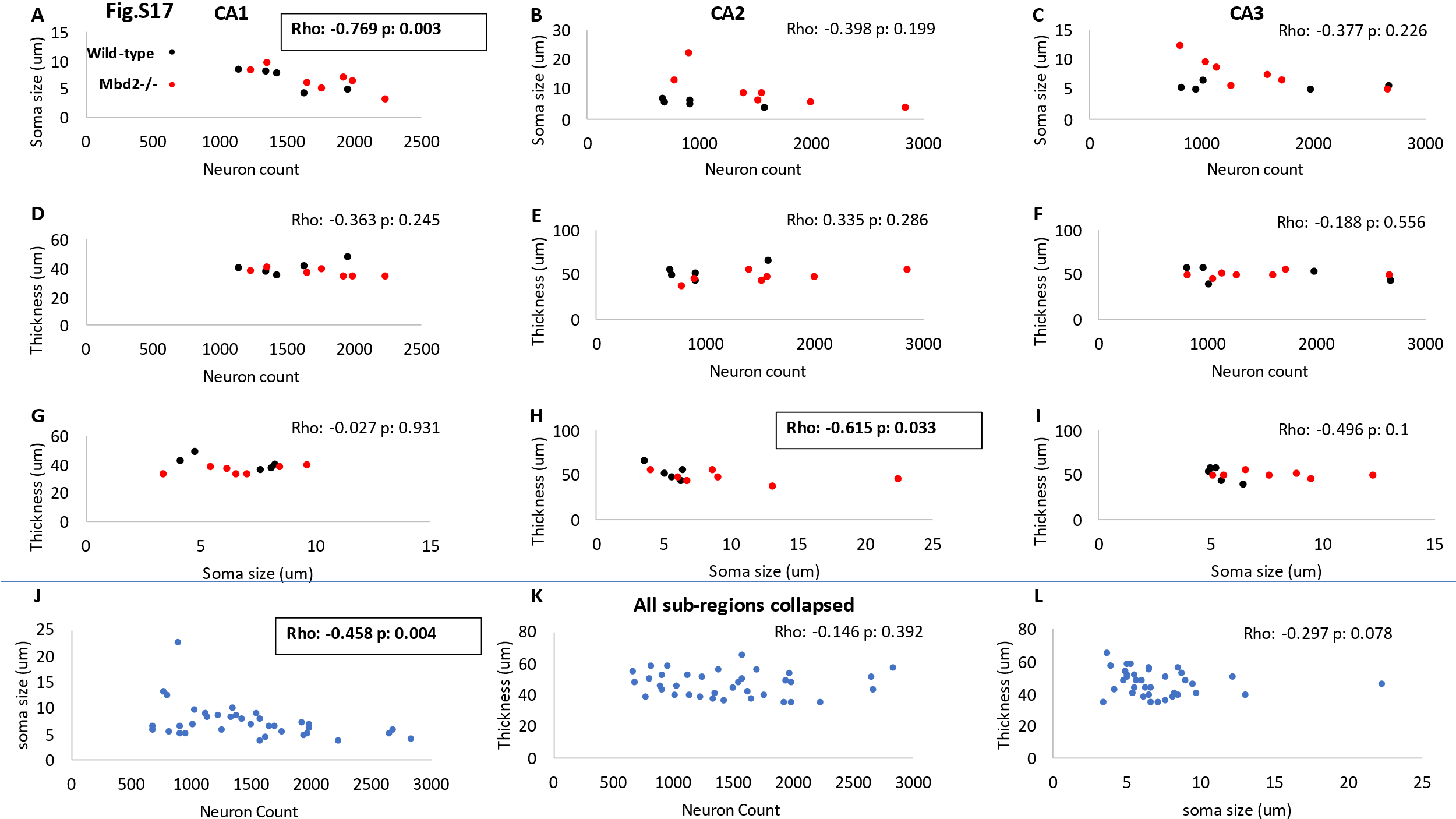


**Supplementary Figure 17.** Correlation analyses of neuron count and neuron soma size (A-C); neuron count and layer thickness (D-F) and neuron soma size and layer thickness (G-I)
of hippocampal subregions CA1-CA3 in wild-type and *Mbd2*-/- mice as determined by quantitative analyses following NeuN immunolabeling. Correlation analyses between neuron count and neuron soma size (J);neuron count and layer thickness (K) and between neuron soma size and layer thickness (L) of the total hippocampus (collapsing CA1, CA2 and CA3 data). Spearman’s Rho for correlation analyses, with exact p-values indicated in each graph.


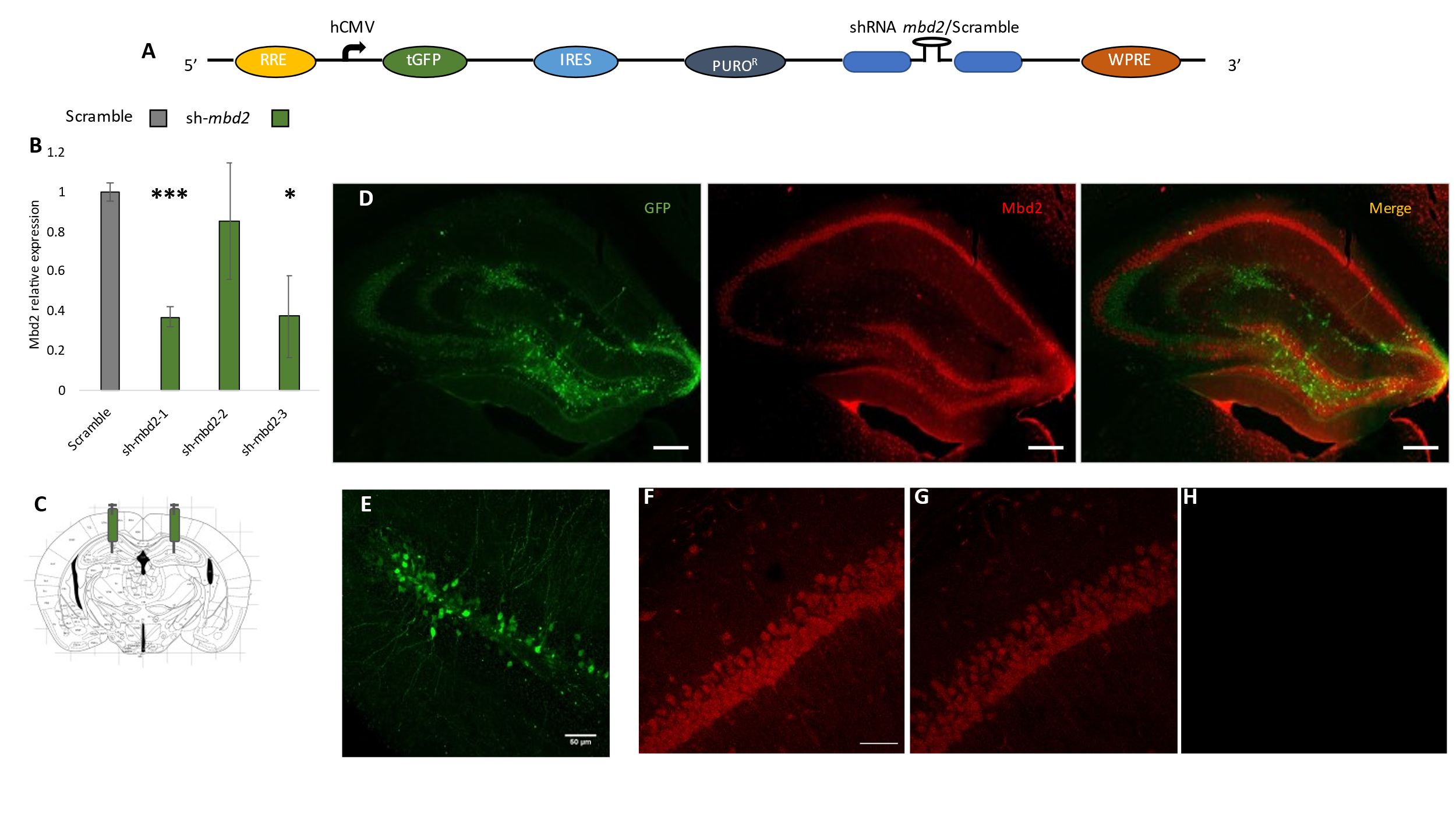


**Supplementary Figure 18.** A. Schematic illustration of the sh-*Mbd2*/scramble constructs. RRE- Rev Response Element, hCMV-human Cytomegalovirus promoter, tGFP-turbo GFP reporter, IRES-Internal Ribosomal Entry Site, PURO^R^-Puromycin Resistance, WPRE-Woodchuck Hepatitis Posttranslational Regulatory Element. B. In-vitro validation of sh-*Mbd2* construct. The effect of sh-*Mbd2* constructs on Mbd2 mRNA levels in lentivirus-treated NIH3T3 was tested by qRT-PCR (change over scramble control). C. Illustration of infusion site adapted from the Paxinos and Franklin mouse brain atlas, and D. confocal fluorescence microscope images showing hippocampus expression of GFP-tagged lentivirus (green), MBD2 (red) and a merged image, scale bar: 200μm; and E. Larger magnification of lentivirus-infected neurons, scale bar: 50 μm. Specificity of the neuronal signal for Mbd2: Mbd2 immunoreactivity (F) was substantially reduced when the primary antibody was pre-incubated with the same gram amount of blocking peptide (1×, G) and completely blocked by an excess of it (5×, H). Scale bar: 50 μm.


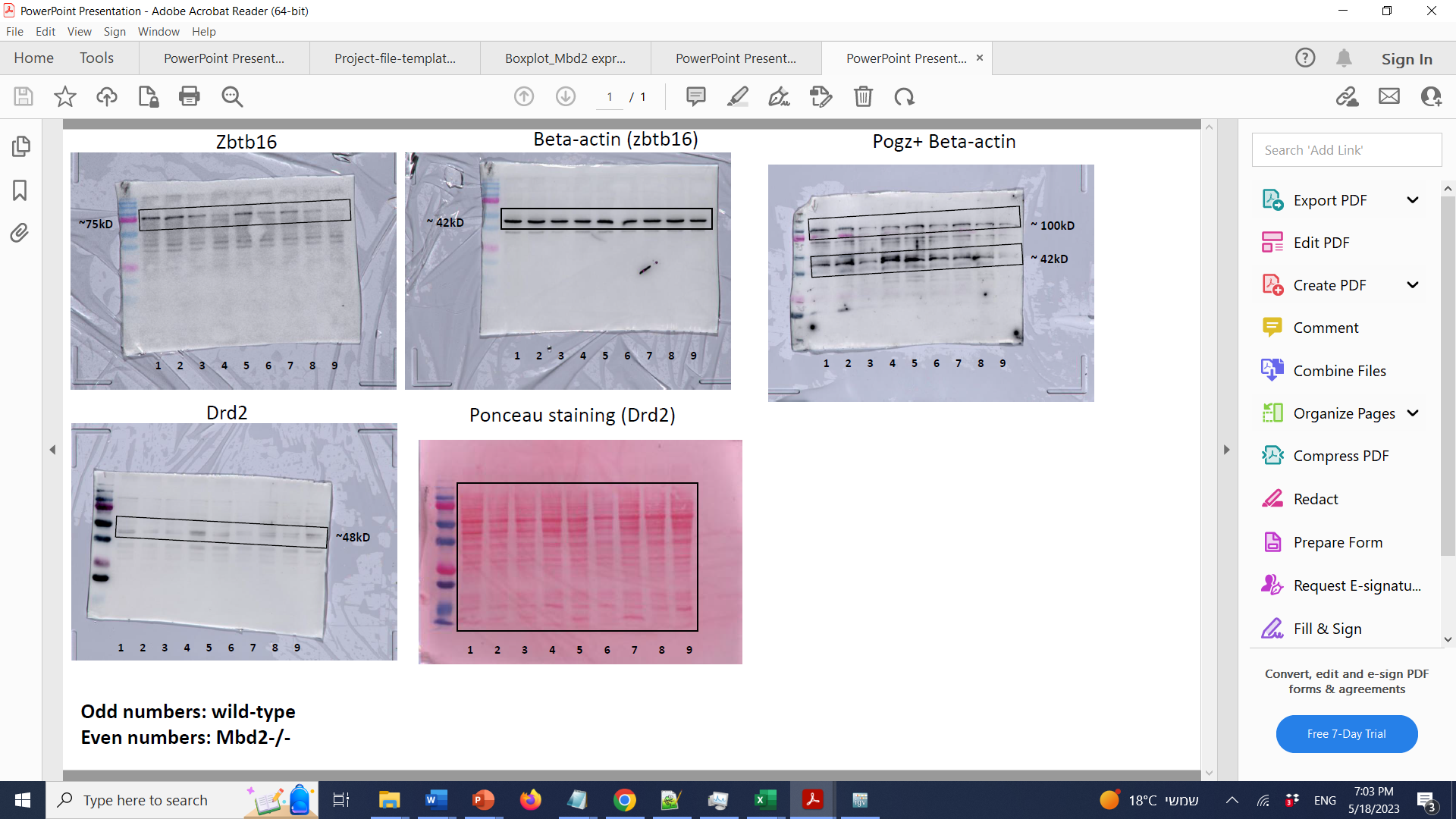


**Supplementary Figure 19.** Uncropped images of immunoblots shown in figure 4E.


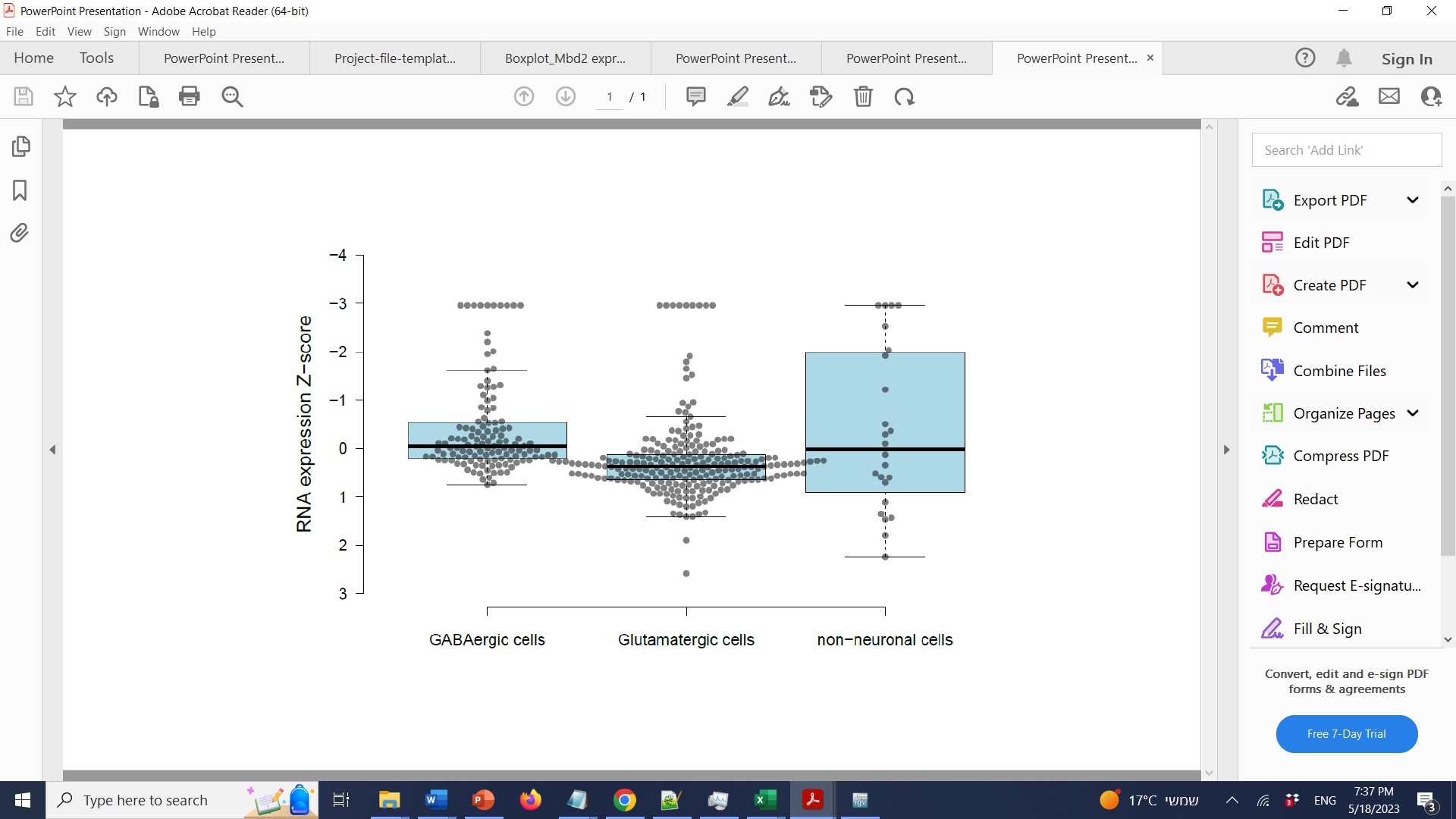


**Supplementary Figure 20.** Single-cell RNA-seq data from the Allen mouse brain atlas was analyzed for Mbd2 mRNA expression levels across cell types within the hippocampus and the cortex. We aggregated the data from 382 cell clusters into three major classes: GABAergic cells, Glutamatergic cells, and non-neuronal cells, and plotted normalized expression levels for each of the 382 cell clusters according to their major class.


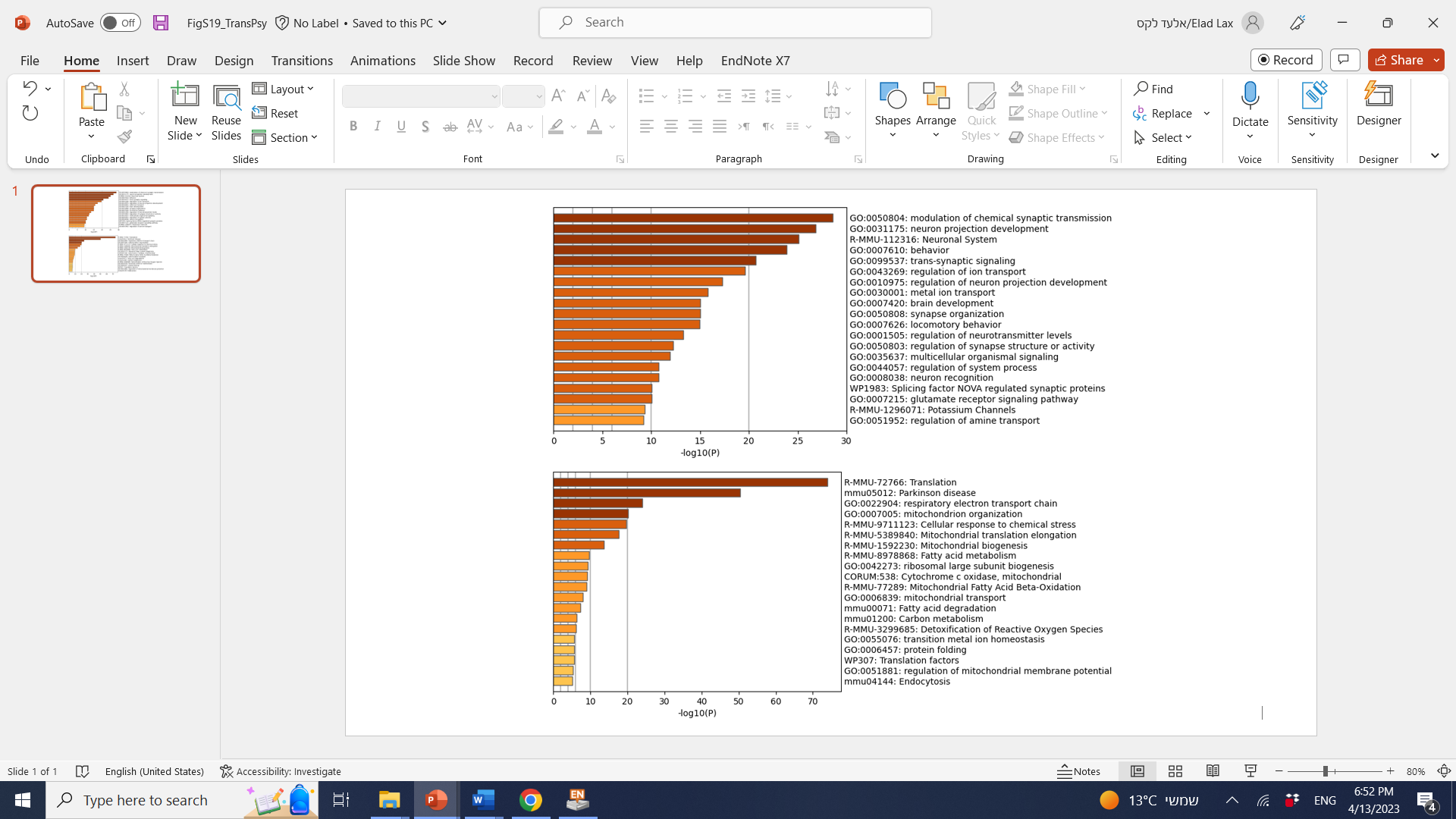

**Supplementary Figure 21.** RNA-seq pathway enrichment analysis using the brain-expressed genes list adapted from <https://www.syngoportal.org/> ^23^ as background gene-list. Upper panel: GO-Pathway analysis enrichment of *Mbd2-/-* down-regulated genes. Upper panel: Gene-network analysis by Metascape of *Mbd2-/-* up-regulated genes.

**References**

1 Gregoire, S., Jang, S. H., Szyf, M. & Stone, L. S. Prenatal maternal stress is associated with increased sensitivity to neuropathic pain and sex-specific changes in supraspinal mRNA expression of epigenetic- and stress-related genes in adulthood. *Behav Brain Res* **380**, 112396, doi:10.1016/j.bbr.2019.112396 (2020).

2 Lax, E. *et al.* PARP-1 is required for retrieval of cocaine-associated memory by binding to the promoter of a novel gene encoding a putative transposase inhibitor. *Mol Psychiatry* **22**, 570-579, doi:10.1038/mp.2016.119 (2017).

3 Kalueff, A. V. *et al.* Neurobiology of rodent self-grooming and its value for translational neuroscience. *Nat Rev Neurosci* **17**, 45-59, doi:10.1038/nrn.2015.8 (2016).

4 Berridge, K. C., Fentress, J. C. & Parr, H. Natural syntax rules control action sequence of rats. *Behav Brain Res* **23**, 59-68, doi:10.1016/0166-4328(87)90242-7 (1987).

5 Heyward, F. D. *et al.* Obesity Weighs down Memory through a Mechanism Involving the Neuroepigenetic Dysregulation of Sirt1. *J Neurosci* **36**, 1324-1335, doi:10.1523/JNEUROSCI.1934-15.2016 (2016).

6 Sato, A. *et al.* Rapamycin reverses impaired social interaction in mouse models of tuberous sclerosis complex. *Nat Commun* **3**, 1292, doi:10.1038/ncomms2295 (2012).

7 Wood, K. H. *et al.* Tagging methyl-CpG-binding domain proteins reveals different spatiotemporal expression and supports distinct functions. *Epigenomics* **8**, 455-473, doi:10.2217/epi-2015-0004 (2016).

8 McNaughton, C. H. *et al.* Evidence for social anxiety and impaired social cognition in a mouse model of fragile X syndrome. *Behav Neurosci* **122**, 293-300, doi:10.1037/0735-7044.122.2.293 (2008).

9 Mineur, Y. S., Huynh, L. X. & Crusio, W. E. Social behavior deficits in the Fmr1 mutant mouse. *Behav Brain Res* **168**, 172-175, doi:10.1016/j.bbr.2005.11.004 (2006).

10 Sams, D. S. *et al.* Neuronal CTCF Is Necessary for Basal and Experience-Dependent Gene Regulation, Memory Formation, and Genomic Structure of BDNF and Arc. *Cell Rep* **17**, 2418-2430, doi:10.1016/j.celrep.2016.11.004 (2016).

11 Langmead, B. Aligning short sequencing reads with Bowtie. *Curr Protoc Bioinformatics* **Chapter 11**, Unit 11 17, doi:10.1002/0471250953.bi1107s32 (2010).

12 Liu, T. Use model-based Analysis of ChIP-Seq (MACS) to analyze short reads generated by sequencing protein-DNA interactions in embryonic stem cells. *Methods Mol Biol* **1150**, 81-95, doi:10.1007/978-1-4939-0512-6_4 (2014).

13 Tarbell, E. D. & Liu, T. HMMRATAC: a Hidden Markov ModeleR for ATAC-seq. *Nucleic Acids Res* **47**, e91, doi:10.1093/nar/gkz533 (2019).

14 Xi, Y. & Li, W. BSMAP: whole genome bisulfite sequence MAPping program. *BMC Bioinformatics* **10**, 232, doi:10.1186/1471-2105-10-232 (2009).

15 Akalin, A. *et al.* methylKit: a comprehensive R package for the analysis of genome-wide DNA methylation profiles. *Genome Biol* **13**, R87, doi:10.1186/gb-2012-13-10-r87 (2012).

16 Heinz, S. *et al.* Simple combinations of lineage-determining transcription factors prime cis-regulatory elements required for macrophage and B cell identities. *Mol Cell* **38**, 576-589, doi:10.1016/j.molcel.2010.05.004 (2010).

17 Thomas-Chollier, M. *et al.* A complete workflow for the analysis of full-size ChIP-seq (and similar) data sets using peak-motifs. *Nat Protoc* **7**, 1551-1568, doi:10.1038/nprot.2012.088 (2012).

18 Lu, J. *et al.* PrimerSuite: A High-Throughput Web-Based Primer Design Program for Multiplex Bisulfite PCR. *Sci Rep* **7**, 41328, doi:10.1038/srep41328 (2017).

19 Szklarczyk, D. *et al.* The STRING database in 2017: quality-controlled protein-protein association networks, made broadly accessible. *Nucleic Acids Res* **45**, D362-D368, doi:10.1093/nar/gkw937 (2017).

20 Zhou, Y. *et al.* Metascape provides a biologist-oriented resource for the analysis of systems-level datasets. *Nat Commun* **10**, 1523, doi:10.1038/s41467-019-09234-6 (2019).

21 Hendrich, B., Guy, J., Ramsahoye, B., Wilson, V. A. & Bird, A. Closely related proteins MBD2 and MBD3 play distinctive but interacting roles in mouse development. *Genes Dev* **15**, 710-723, doi:10.1101/gad.194101 (2001).

22 Sanders, S. J. *et al.* Insights into Autism Spectrum Disorder Genomic Architecture and Biology from 71 Risk Loci. *Neuron* **87**, 1215-1233, doi:10.1016/j.neuron.2015.09.016 (2015).

23 Koopmans, F. *et al.* SynGO: An Evidence-Based, Expert-Curated Knowledge Base for the Synapse. *Neuron* **103**, 217-234 e214, doi:10.1016/j.neuron.2019.05.002 (2019).
